# EZH2 inhibition sensitizes retinoic acid-driven senescence in synovial sarcoma

**DOI:** 10.1038/s41419-024-07176-6

**Published:** 2024-11-16

**Authors:** Muhammad Mushtaq, Judit Liaño-Pons, Jiansheng Wang, Mohammad Alzrigat, Ye Yuan, María Victoria Ruiz-Pérez, Yi Chen, Elena Kashuba, Felix Haglund de Flon, Bertha Brodin, Marie Arsenian-Henriksson

**Affiliations:** 1 Department of Microbiology, Tumor and Cell Biology (MTC), Biomedicum, Karolinska Institutet, SE-171 65, Stockholm, Sweden; 2 Department of Oncology-Pathology, Karolinska Institutet, Solna, SE-171 76 Stockholm Sweden; 3 Division of Hematology and Oncology, Department of Medicine, Columbia Stem Cell Initiative, Columbia University Irving Medical Center, New York, USA; 4 RE Kavetsky Institute of Experimental Pathology, Oncology and Radiobiology of NAS of Ukraine, 03022 Kyiv, Ukraine; 5 Department of Applied Physics, Biomedical and X-Ray Physics, KTH Royal Institute of Technology, SE-10691 Stockholm, Sweden; 6 Present Address: Department of Biotechnology, Faculty of Life Sciences and Informatics. Balochistan University of Information Technology, Engineering, and Management Sciences (BUITEMS), 87300 Quetta, Pakistan

**Keywords:** Sarcoma, Senescence

## Abstract

Synovial sarcoma (SS) is driven by a unique t(18;X) chromosomal translocation resulting in expression of the SS18-SSX fusion oncoprotein, a transcriptional regulator with both activating and repressing functions. However, the manner in which SS18-SSX contributes to the development of SS is not entirely known. Here, we show that SS18-SSX drives the expression of Preferentially Expressed Antigen in Melanoma (PRAME), which is highly expressed in SS but whose function remains poorly understood. The fusion protein directly binds and activates the *PRAME* promoter and we found that expression of SS18-SSX and PRAME are positively correlated. We provide evidence that PRAME modulates retinoic acid (RA) signaling, forming a ternary complex with the RA receptor α (RARα) and the Enhancer of Zeste Homolog 2 (EZH2). Knockdown of PRAME suppressed the response to all-*trans* retinoic acid (ATRA) supporting PRAME’s role in modulating RA-signaling. Notably, we demonstrate that combined pharmacological inhibition of EZH2 and treatment with ATRA reconstituted RA signaling followed by reduced proliferation and induction of cellular senescence. In conclusion, our data provides new insights on the role of the SS18-SSX fusion protein in regulation of PRAME expression and RA signaling, highlighting the therapeutic potential of disrupting the RARα-PRAME-EZH2 complex in SS.

**Figure:**
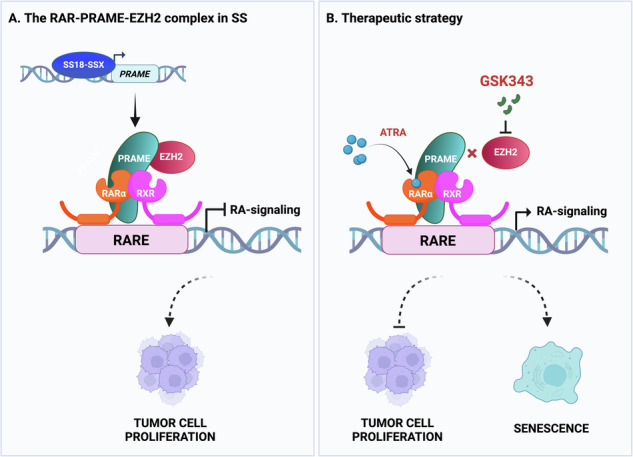
**Schematic presentation of the proposed model**. **A The RARα-PRAME-EZH2 ternary complex in SS**. The fusion SS18-SSX oncoprotein binds to the *PRAME* promoter and activates its expression. PRAME in turn interacts with RARα-RXR heterodimers as well as with EZH2, and the complex binds to retinoic acid response elements (RAREs) in the DNA. This results in transcriptional repression of retinoic acid (RA) responsive genes and thus inhibition of RA-signaling, allowing tumor cell proliferation. **B Therapeutic strategy**. Treatment with an EZH2 inhibitor, such as GSK343, or activation of RAR receptors via all-*trans* retinoic acid (ATRA), disrupts the RARα-PRAME-EZH2 ternary complex and restores RA-signaling. Exposure to GSK343 or ATRA results in inhibition of cell proliferation and induction of cellular senescence, where GSK343 shows a dominant effect. The Figure was created with Biorender.com.

**Schematic presentation of the proposed model**. **A The RARα-PRAME-EZH2 ternary complex in SS**. The fusion SS18-SSX oncoprotein binds to the *PRAME* promoter and activates its expression. PRAME in turn interacts with RARα-RXR heterodimers as well as with EZH2, and the complex binds to retinoic acid response elements (RAREs) in the DNA. This results in transcriptional repression of retinoic acid (RA) responsive genes and thus inhibition of RA-signaling, allowing tumor cell proliferation. **B Therapeutic strategy**. Treatment with an EZH2 inhibitor, such as GSK343, or activation of RAR receptors via all-*trans* retinoic acid (ATRA), disrupts the RARα-PRAME-EZH2 ternary complex and restores RA-signaling. Exposure to GSK343 or ATRA results in inhibition of cell proliferation and induction of cellular senescence, where GSK343 shows a dominant effect. The Figure was created with Biorender.com.

## Introduction

Synovial sarcoma (SS) is a rare and malignant mesenchymal tumor affecting adolescents and young adults, with a mortality rate of ~50% within ten years of diagnosis despite multimodal therapies [1]. The main cytogenetic event is a balanced chromosomal translocation t(X;18)(p11.2;q11.2) [2–4], resulting in fusion of the *synovial sarcoma translocation, chromosome 18* (*SS18*) gene, encoding one member of the switch/sucrose-non-fermentable (SWI/SNF) complex, with one of the *synovial sarcoma, X-breakpoint 1, 2*, or *4* (*SSX1, SSX2*, or *SSX4*) genes, which encode transcriptional repressors [5, 6]. Notably, the sole expression of SS18-SSX1/SSX2 is sufficient to drive SS genesis in a mouse model, demonstrating the oncogenic capacity of t(X;18)(p11.2;q11.2) fusion products [7–10].

The Preferentially Expressed Antigen in Melanoma (PRAME) is a cancer-testis antigen with roles in cell proliferation, apoptosis, differentiation, and metastasis. Its expression is restricted to somatic tissues but upregulated in various cancers [11–15], likely due to promoter hypomethylation [16, 17]. PRAME is a dominant repressor of retinoic acid (RA) signaling in melanoma and acute myeloid leukemia (AML) by interaction with the Enhancer of Zeste Homolog 2 (EZH2) and the RA receptor α (RARα) at RA response elements (RAREs) [18, 19]. The histone methyltransferase EZH2 is a catalytic subunit of the Polycomb repressor complex 2 (PRC2) [20] and as such catalyzes tri-methylation of histone H3 at Lys27 (H3K27me3), resulting in silencing of target genes [21, 22]. Importantly, EZH2 plays a critical role in tumor progression and metastasis and is aberrantly expressed in different malignancies [23, 24]. The discovery of highly specific EZH2 inhibitors during the past decade has demonstrated its therapeutic potential as target for cancer treatment [25]. Retinoic acid regulates numerous biological processes by serving as ligand for the nuclear RA receptors (RARα, RARβ, and RARγ) as well as the retinoid X receptors (RXRs) [26]. These dimers regulate transcription of genes involved in development, tissue homeostasis, cell cycle, differentiation, senescence, and cell death [27, 28]. Thus, inhibition of RA signaling can enable cancer cells to bypass anti-tumor responses [29].

Here, we show that PRAME is highly expressed in SS in comparison to other soft tissue sarcomas (STS) and is directly regulated by SS18-SSX, presenting a mechanism by which this fusion protein can drive oncogenesis. PRAME in turn forms a ternary complex with EZH2 and RARα which disrupts RA signaling. Our data reveal that either EZH2 inhibition or RA treatment restores RA-signaling, leading to induction of senescence, suggesting a potential novel approach for treatment of SS.

## Materials and methods

Please see Supplementary Information.

## Results

### Expression pattern of *PRAME, EZH2*, and *RARs* in soft tissue sarcomas

**Fig. 1:**
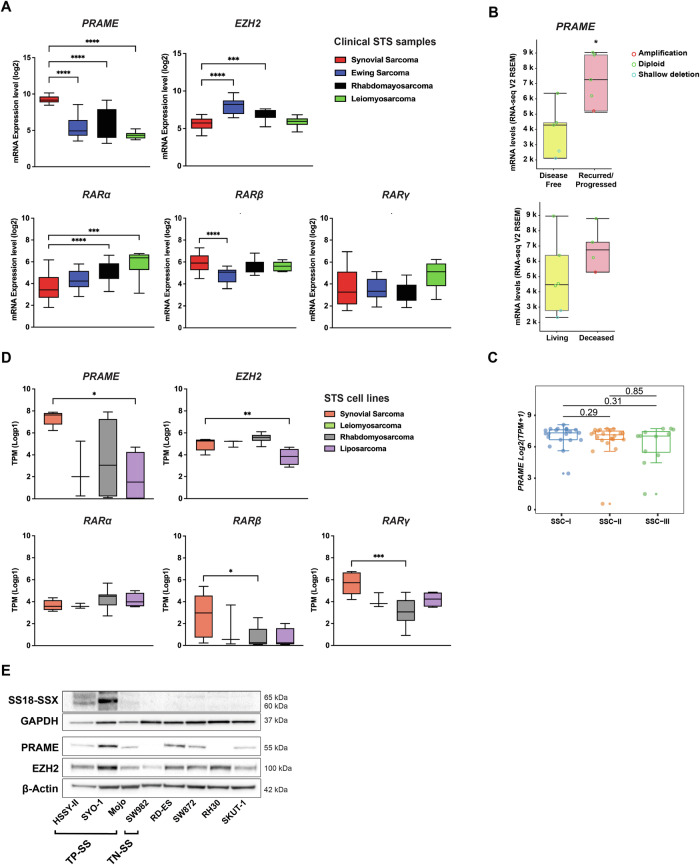
Expression of *PRAME*, *EZH2*, and *RARs* in soft tissue sarcomas. **A** In silico mRNA expression analysis in patient STS samples. Log2 values of mRNA gene expression of *PRAME, EZH2*, as well as *RARα, β, and γ* from the Boshoff and Filion datasets [71, 72] were downloaded from the R2 Genomic Platform and applied to GraphPad Prism. Statistical analysis: one-way ANOVA with multiple comparison test. **B** Correlation of *PRAME* mRNA levels with clinical parameters. The cBioportal platform was used to analyze SS data from the TCGA adult STS cohort (*n* = 10). Presentation of the correlation with “disease free status” (upper panel) and “overall survival status” (lower panel). Copy number variations in *PRAME* are shown in red (amplification), green (diploid), or blue (shallow deletion) circles for each respective patient. Statistical analysis: unpaired *t*-test. **C** *PRAME* expression in biological subgroups. *PRAME* levels in Log2(TPM + 1) from the Chen cohort (*n* = 55) classified in three SS clusters (SSC): SSC-I (poorly differentiated), SSC-II (monophasic), and SSC-III (biphasic). Statistical analysis: Mann-Whitney U test. **D** In silico mRNA expression analysis in STS cell lines. The Log *p*-values of mRNA gene expression of *PRAME*, *EZH2*, as well as *RARα*, *β*, and *γ* were downloaded from the Cell Line Encyclopedia (Depmap portal) and applied to GraphPad Prism. Statistical analysis: one-way ANOVA with multiple comparison test. **E** Expression of SS18-SSX, PRAME, and EZH2 in STS cells. The SS18-SSX, PRAME and EZH2 levels were analyzed by Western blot in a panel of STS cell lines. GAPDH and β-Actin were used as loading controls. Molecular weight markers in kDa are shown to the right. Translocation positive SS (TP-SS; SYO-1, HSSY-II, MoJo), translocation negative SS (TN-SS; SW982), Ewing Sarcoma (RD-ES), rhabdomyosarcoma (RH-30), liposarcoma (SW872), and leiomyosarcoma (SKUT-1) as indicated. One representative from three independent experiments is shown. Uncropped Western blots are presented in Original data with quantifications in Fig. S1B.

Previous studies on high PRAME expression in SS, and reports on its interaction with EZH2 and RARs in melanoma and AML [18, 19] prompted us to analyze the expression of *PRAME, EZH2*, and the *RAR* genes in different soft-tissue sarcomas (STS). Using the R2 platform, we found significantly higher *PRAME* expression in SS tissue compared to specimens from Ewing sarcoma, rhabdomyosarcoma, or leiomyosarcoma, while it did not differ among the latter STS (Fig. 1A). Expression of *EZH2* and the *RAR* genes was either similar or lower in SS compared to other STS, except for *RARβ*, which showed higher levels in SS compared to Ewing sarcoma (Fig. 1A). Analysis using the COSMIC database demonstrated that the SS samples (*n* = 60) carried an intact *PRAME* gene without any gene copy number variation (Supplementary Material and Methods, “mRNA Expression Analysis of PRAME in STS”). When analyzing the correlation between *PRAME* and clinical parameters in another SS cohort (*n* = 10) [30], we observed high levels in tumors from recurred/progressed *versus* disease-free cases as well as from deceased *versus* living patients, although the latter was not statistically significant (Fig. 1B). In addition, *PRAME* expression in the Chen cohort (*n* = 55) [31] was divided in three recently reported clusters: SSC-I (poorly differentiated), SSC-II (monophasic), and SSC-III (biphasic) [31]. We found that overall and metastasis-free survival were not significantly different among tumors with high *versus* low *PRAME* expression (Fig. S1A), but *PRAME* levels were slightly higher in SSC-I, although not significant (Fig. 1C).

Next, we performed in silico gene expression analysis of *PRAME, EZH2*, and the *RAR* genes in synovial, leiomyosarcoma, rhabdomyosarcoma, and liposarcoma STS cell lines. The mRNA expression pattern was in line with tumor data showing high *PRAME* expression in SS compared to other STS (Fig. 1D). This was specific for *PRAME*, although we also observed some differences in *EZH2* and the *RAR* expression levels between cell lines. Western blot analyses of SS18-SSX, PRAME, and EZH2 showed PRAME expression in translocation positive SS (TP-SS) and in other STS cells, including the RD-ES Ewing sarcoma cell line, while the SW982 translocation negative (TN-SS) cells had no detectable expression. The EZH2 levels were similar in TP-SS compared to other STS cells (Figs. 1E and S1B).

**Fig. 2:**
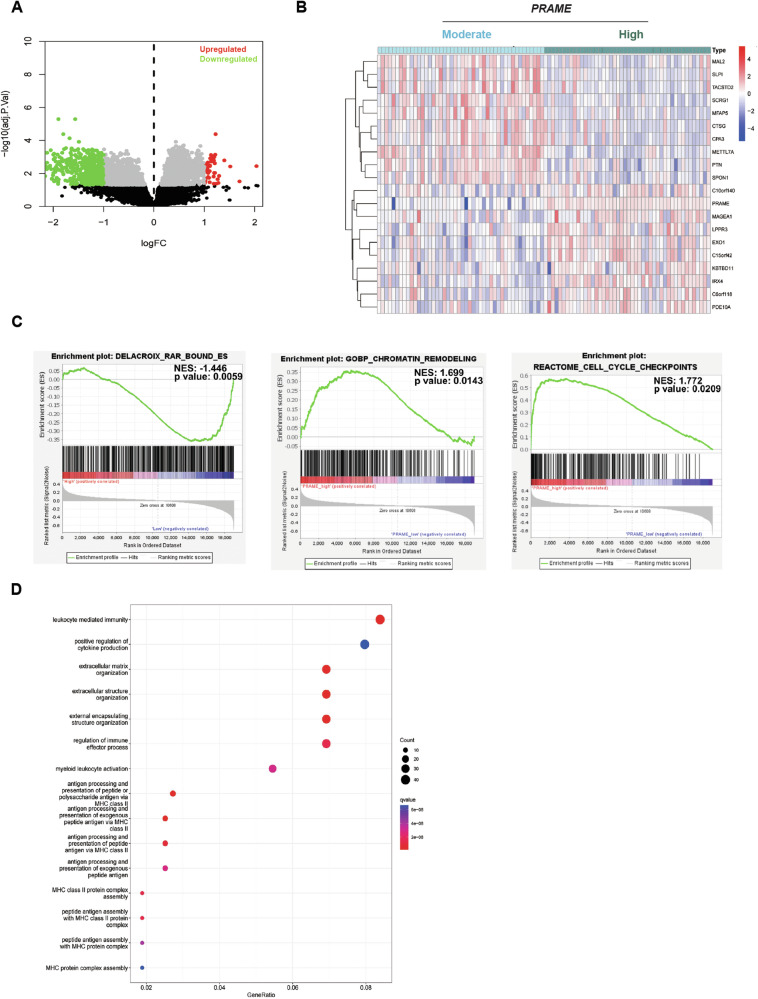
*PRAME*-related gene expression analysis in SS. Data was extracted from the GSE40018 and GSE40021 datasets. **A** Volcano blot shows gene expression analysis in SS specimens correlating with expression of *PRAME*. The x-axis shows the fold change (log) of gene expression normalized to *PRAME* while the y-axis presents adjusted *p*-values. Genes negatively (green) and positively (red) correlated to *PRAME* expression are presented. **B** Heatmap of the top twenty differentially expressed genes. Data was divided into *PRAME* ^high^ (dark green) and *PRAME* ^moderate^ (light blue) expression with the median expression score as a cutoff. Differential gene expression analysis was performed using the limma package. **C** Gene Set Enrichment Analysis (GSEA) plots. GSEA for RAR-bound genes, Chromatin Remodeling genes, and Cell Cycle checkpoint genes using the DELACROIX_RAR_BOUND_ES.gmt, GOBP_CHROMATIN_REMODELING.gmt, the REACTOME_CELL_CYCLE_CHECKPOINTS.gmt, and the gene sets in the GSEA Molecular Signature Database are presented as indicated. The “Signal-to-Noise” ratio (SNR) statistic was used to rank genes per correlation with *PRAME* ^high^ (red) and *PRAME* ^moderate^ (blue) expression. Each green curve corresponds to the enrichment score (ES) curve, which is the running sum of the weighted enrichment score obtained from the GSEA software. The normalized enrichment score (NES) and the adjusted *p*-values are indicated within each graph. **D** Gene Ontology (GO) pathway analysis. Enrichment analysis was performed using the clusterProfiler packages where the y-axis represents clustered GO terms and the x-axis the GeneRatio i.e. the ratio of the number of genes enriched in one GO term to the number of upregulated or downregulated DEGs, respectively.

We explored PRAME-related gene expression in two SS patient datasets (GSE40018, GSE40021), and identified 576 differentially expressed genes ( | logFC | > 1 and padj < 0.05) between *PRAME* ^high^ and *PRAME* ^moderate^ tumors (Fig. 2A). Briefly, 49 genes showed a significantly positive correlation while 301 genes were negatively correlated with *PRAME*. The top twenty differentially expressed genes were depicted in a heatmap (Fig. 2B). Gene Set Enrichment Analysis (GSEA) showed that *PRAME* expression was negatively correlated with the expression of RAR-target genes (Fig. 2C left panel). We further identified biological processes positively correlated with *PRAME* expression including ribosome biogenesis, DNA repair, chromatin remodeling, RNA polymerase I transcription initiation, cell cycle checkpoints, and regulation of p53 activity (Figs. 2C and S2; Table S1). Gene Ontology (GO) (Fig. 2D) and Kyoto Encyclopedia of Genes and Genomes (KEGG) analysis (Fig. S3) revealed that a majority of the differentially expressed genes were involved in immune response processes. In summary, our data demonstrated that high PRAME levels are a characteristic of both TP-SS patient samples as well as cell lines, affecting several key pathways, which might result in deregulation of cellular homeostasis.

### The SS18-SSX fusion protein binds the *PRAME* promoter region and drives its expression

**Fig. 3:**
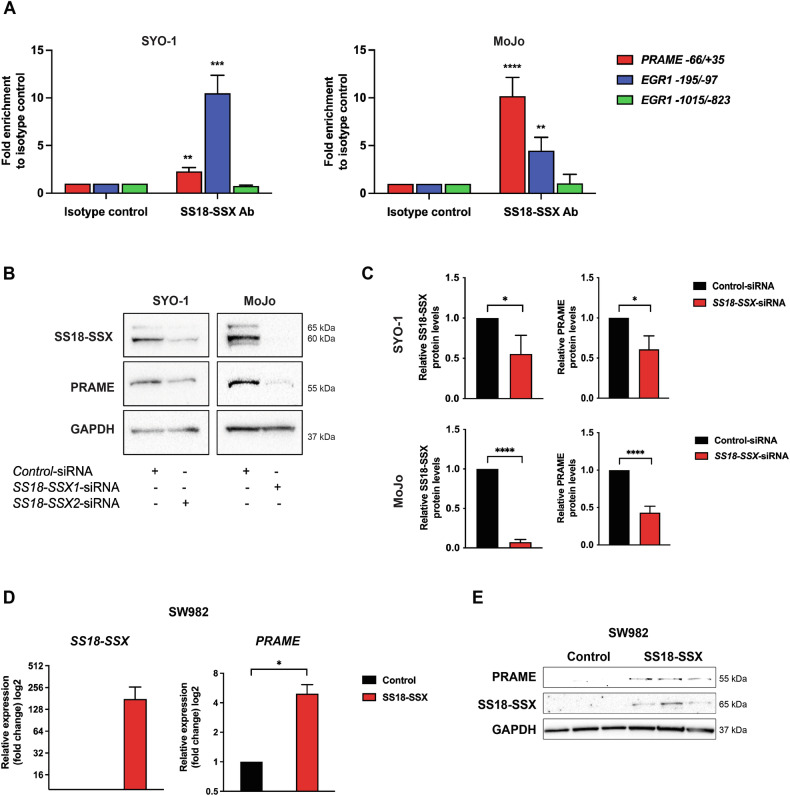
SS18-SSX targets the *PRAME* promoter and induces its expression. **A** Chromatin immunoprecipitation analysis of SS18-SSX binding to the *PRAME* promoter. Chromatin immunoprecipitation followed by qPCR performed in SYO-1 (left panel) and MoJo (right panel) cells demonstrating binding of SS18-SSX to the *PRAME*^*-66/+35*^ promoter region (red). *EGR1*^*-195/-97*^ was used as positive (blue) while EGR1^*-1015/-823*^ was employed as negative control (green). Data represents results from three to four independent experiments, presented in Original data. Statistical analysis: unpaired *t*-test. **B** Effect of SS18-SSX knockdown on PRAME expression. SYO-1 and MoJo cells were treated with control siRNA or siRNA against *SS18-SSX2* or *SS18-SSX1*, respectively, as indicated. After confirmation of knockdown, expression of PRAME was analyzed using Western blot. GAPDH was used as a loading control. Molecular weight markers in kDa are shown to the right. The uncropped blots are presented in Original data. **C** Quantification of experiment in **B**, showing SS18-SSX and PRAME levels relative to GAPDH. Statistical analysis: unpaired *t*-test. **D** Expression of *PRAME* upon ectopic expression of SS18-SSX in SW982 cells. The translocation negative SW982 SS cells were transfected with a control vector or an expression construct carrying the *SS18-SSX1* gene. After three days the relative mRNA expression of *SS18-SSX* (left panel) and *PRAME* (right panel) was analyzed using *GAPDH* as endogenous control. Three independent experiments were performed, and unpaired *t*-test analysis was applied. Raw values are found in Original data. **E** Expression of SS18-SSX and PRAME in SW982 cells. Proteins as indicated were analyzed by Western blot one week post transfection with a SS18-SSX expressing plasmid. GAPDH was used as a loading control. Molecular weight markers in kDa are indicated to the right. The blot shows three independent repeats of control and SS18-SSX overexpression experiments. Uncropped blots are presented in Original data.

In search for a mechanism responsible for the high PRAME expression in TP-SS, we interrogated whether SS18-SSX could directly regulate *PRAME* transcription. To this end, we performed chromatin immunoprecipitation (ChIP) assays of SS18-SSX, followed by qPCR in both SYO-1 and MoJo cells. Our results showed a significant enrichment of SS18-SSX at −66/ + 35 bp from the transcription start site in the *PRAME* promoter (Fig. 3A). As positive control we used the early upstream region (*-195/-97*) of the *EGR1* promoter to which SS18-SSX has been shown to bindand the *EGR1*^-1015/-823^ region as negative control [32, 33].

To explore whether this binding affects PRAME expression, we downregulated *SS18-SSX* in SYO-1 and MoJo cells using siRNAs. Interestingly, we observed a significant reduction in PRAME levels following SS18-SSX knockdown in both cell lines (Fig. 3B, C). As a reverse experiment, SS18-SSX was ectopically expressed in SW982 cells, a unique SS cell line lacking both the fusion oncoprotein as well as PRAME expression (Fig. 1E). We verified PRAME expression at both mRNA and protein level in cells expressing SS18-SSX1 but not in control cells (Fig. 3D, E). We conclude that SS18-SSX directly binds to the *PRAME* promoter and induces its expression at the transcriptional level.

### PRAME interacts with RARα and EZH2 in SS cell lines

**Fig. 4:**
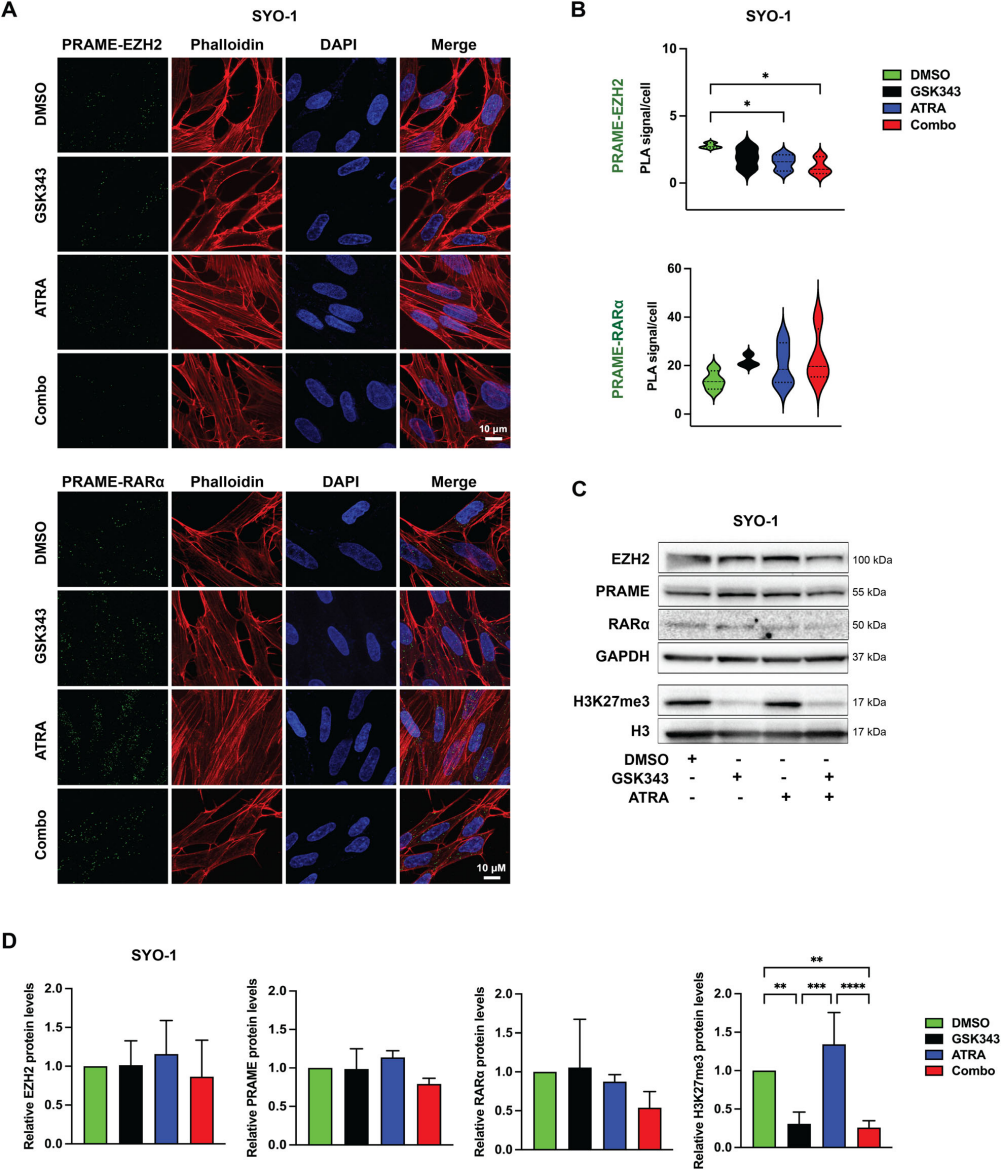
Effect of GSK343 and/or ATRA on the RARα-PRAME-EZH2 ternary complex. **A** Proximity ligation assay (PLA)-based analysis of PRAME-EZH2 (upper panel) or PRAME-RARα (lower panel) colocalization in SYO-1 cells treated with DMSO, 2.5 µM GSK343, 5 µM ATRA individually or in combination for 72 h. PLA signal (green) and nuclei (DAPI; blue). **B** Quantification of the PLA signal/cell. The violin plots represent data from at least three independent experiments. For statistical analysis one-way ANOVA with multiple comparison test was performed. **C** Protein expression by Western blot. Expression of EZH2, PRAME, RARα, and H3K27me3 in SYO-1 after 72 h treatment as indicated. GAPDH and total H3 were used as loading controls, respectively. Molecular weight markers in kDa are shown to the right. Representative blots from three independent experiments are shown. Images of the uncropped scans are presented in Original data. **D** Quantification of the Western blot in **C**. Protein levels from three independent experiments were normalized against the loading control and represented as fold change to the control (DMSO). For statistical analysis one-way ANOVA with multiple comparison test was performed.

Our in silico analysis of mRNA expression in two SS patient cohorts suggested an inverse correlation between PRAME and RAR-signaling (Fig. 2C). Following previous findings demonstrating PRAME as a negative regulator of RA signaling by forming a ternary complex with EZH2 and RARα in melanoma cells [18], we employed proximity ligation assay (PLA) to study PRAME-EZH2 and PRAME-RARα interactions in SYO-1 and MoJo cells and found that PRAME physically interacts with EZH2 and RARα as shown by the green PLA signals (Figs. 4A, B and S4A, B). As PRAME inhibitors are not available, we evaluated the effects of the EZH2 inhibitor (EZH2i) GSK343 and all-*trans* retinoic acid (ATRA) in single and combination treatment conditions on PRAME-EZH2 as well as PRAME-RARα interactions. Our PLAs demonstrated that the PRAME-EZH2 dimer was disrupted by the combination treatment in both cell lines, as well as by ATRA alone in SYO-1 cells (Fig. 4B). In contrast to a previous study [18], we did not observe significant differences in PRAME-RARα complexes upon ATRA or GSK343 treatment (Figs. 4B and S4B). The observed changes were not due to decreased protein levels, as no significant changes were revealed by Western blot. Analysis of H3K27me3 confirmed the specificity of GSK343 by the reduction of this mark without affecting total H3 levels (Figs. 4C, D and S4C, D). Together, our data show that PRAME forms complexes with EZH2 and RARα in SS cells, and that combined treatment with GSK343 and ATRA disrupts the PRAME-EZH2 interaction.

### Reduced SS cell proliferation upon EZH2 inhibition and ATRA treatment

**Fig. 5:**
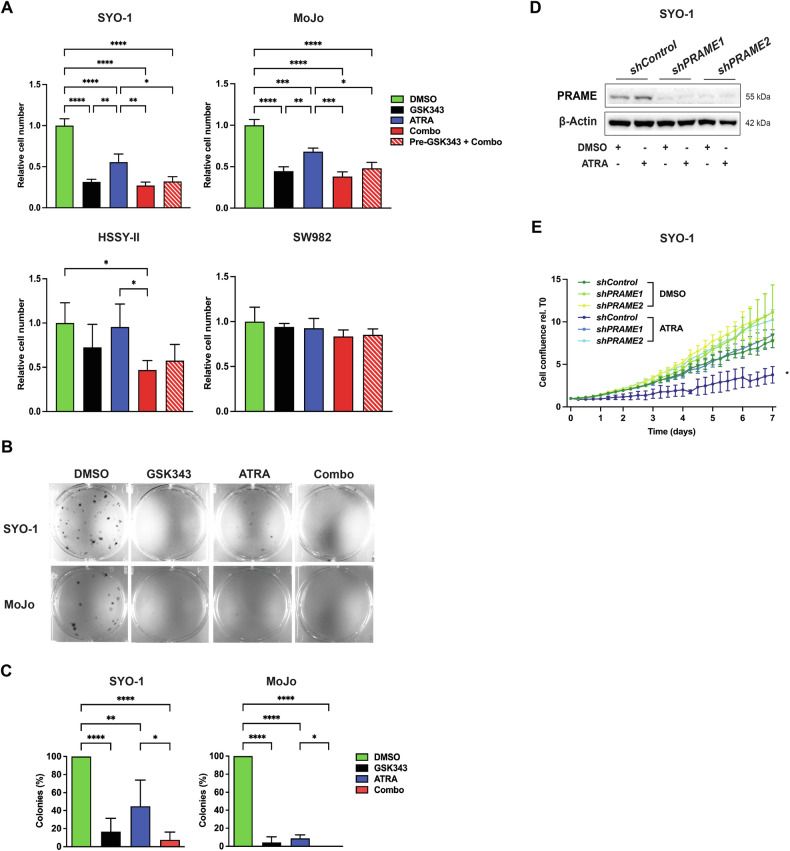
Effect of RARα-PRAME-EZH2 on the proliferation of SS cells. **A** Cell viability assay. MoJo, SYO-1, HSSY-II, and SW982 cells were incubated with DMSO, 2.5 µM GSK343, or 5 µM ATRA, alone or in combination for seven days. In “Pre-GSK343 + Combo”, cells were treated with GSK343 for three days, then switched to Combo exposure up to day seven followed by manual counting. Relative cell number to DMSO is presented from at least three independent experiments. Statistical analysis: one-way ANOVA with multiple comparison test. **B** Colony formation assay. 200 cells per well were cultured in six-well plates and treated the next day with DMSO, 2.5 µM GSK343, 5 µM ATRA, or the combination with continued treatment for two (SYO-1) or four (MoJo) weeks followed by crystal violet staining. One representative from five independent experiments is shown. **C** Quantification of the data in **B**. The number of colonies were counted manually, and the percentage of colonies to control cultures are presented. The bars represent standard deviation of the mean of colonies in each culture. Statistical analysis: one-way ANOVA with multiple comparisons test. **D** PRAME protein levels following ectopic overexpression. Analysis of cells transduced with lentiviral particles carrying a control vector or either of two constructs with shRNAs against *PRAME* as indicated. Cells were treated with DMSO or 5 µM ATRA for three days. β-Actin was used as a loading control and molecular weight markers in kDa are shown to the right. Representative blots from three independent experiments are shown. Uncropped blots and quantifications are presented in Original data and Fig. S7B, respectively. **E** Response of PRAME knockdown cells to ATRA. SYO-1 *shControl*, *shPRAME1*, and *shPRAME2* cells were treated with DMSO or 5 µM ATRA for seven days and assessed using IncuCyte live-cell proliferation analysis. Confluence was normalized *versus* the first time-point (0 h) and presented as the mean of three independent experiments. Statistical analysis: multiple unpaired *t*-tests.

To analyze whether targeting the RAR-PRAME-EZH2 axis has anti-tumor activity, we assessed cell proliferation in SYO-1, MoJo, and HSSY-II (TP-SS) and SW982 (TN-SS) cells following single treatment with GSK343 or ATRA, and in combination for seven days. Single GSK343 or ATRA profoundly affected cell proliferation in SYO-1 and MoJo while slightly in HSSY-II cells and the combination did not further inhibit proliferation when compared to GSK343 alone (Fig. 5A). We did not detect differences between pre-treating with GSK343 for three days followed by combination treatment (“Pre-GSK343+Combo”) compared to simultaneous treatment during seven days (“Combo”). Notably, the PRAME-negative SW982 TN-SS cells were more resistant to both GSK343 as well as ATRA compared to the TP-SS SYO-1 cells, as also shown by the higher IC_50_ values as assessed by BrdU assays (Figs. 5A and S5A). We further studied cell growth at shorter time-points by cell counting at 72 h (Fig. S5B) and MTT assay following 24, 48, and 72 h (Fig. S5C) and found that cell proliferation and viability were mainly affected by combination treatment at 72 h in all TP-SS cell lines when compared to DMSO control treated cells (Fig. S5B). Consistent with these observations, we found a marked decrease in colony formation following ATRA or GSK343 incubation in TP-SS cells, while no effect was observed in SW982 cells. In this assay, GSK343 exerted a dominant effect, overriding the influence of ATRA when in combination in SYO-1 and MoJo but not in HSSY-II cells, where the combination was more effective in reducing colony numbers (Figs. 5B, C and S5D). To analyze any possible synergy between GSK343 and ATRA, we used different concentrations of both compounds at 72 h as well as seven days and indeed observed synergy in SW982 at 72 h while additive effects in SYO-1 cells (Fig. S6A, B).

To examine whether the anti-proliferative effects detected upon reconstitution of RA signaling are PRAME-dependent, we generated SYO-1 cells carrying either of two specific shRNAs against *PRAME* or a scrambled shRNA as control. As expected, both *shPRAME1* and *shPRAME2* cells showed lower levels of PRAME than *shControl* cells as assessed by Western blot (Figs. 5D and S7B) and cell proliferation was significantly reduced in *shControl* whereas *shPRAME* cells were unaffected upon ATRA treatment (Fig. 5E). In line, the RA target and cell cycle inhibitor p21 [18] was robustly increased in ATRA-treated *shControl* but not in *PRAME* knockdown cells, while the latter showed high p21 levels whether treated or not (Fig. S7A, B). Collectively, our results indicated that PRAME-positive SS cells were sensitive to reconstitution of RA signaling, while PRAME-negative (i.e. SW982 cells) and *PRAME-*knockdown cells were ATRA resistant.

### Cumulative effect of ATRA and GSK343 on reconstitution of RA signaling and induction of senescence

**Fig. 6:**
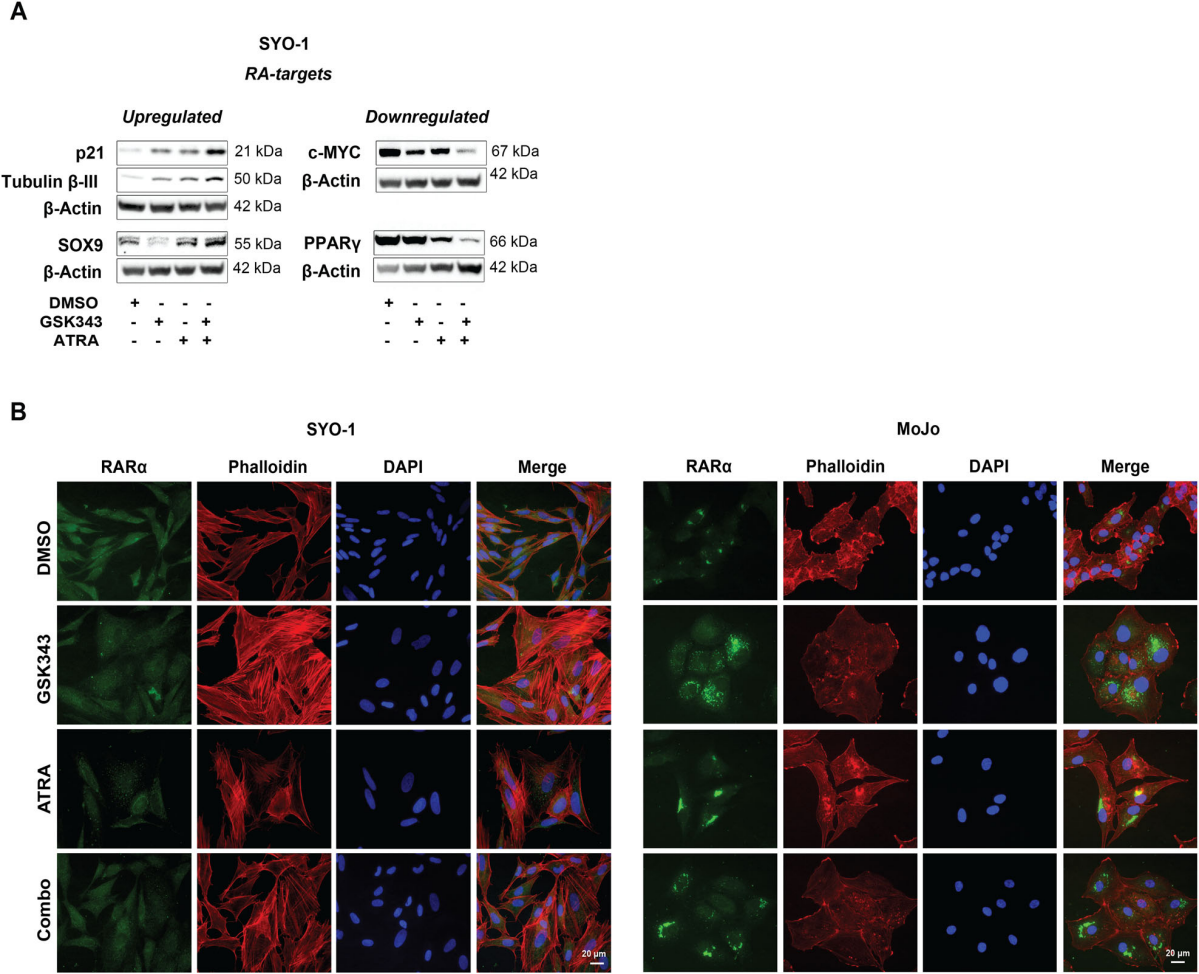
Effect of GSK343 and ATRA on RA-driven signaling pathways. **A** Changes of RA targets at the protein level. SYO-1 cells were treated with DMSO, 5 µM ATRA, 2.5 µM GSK343, or the combination. The expression of p21, Tubulin-βIII, and PPARγ was analyzed by Western blot after three days while the levels of c-MYC and SOX9 were assessed after seven days. β-Actin was used as a loading control. Molecular weight markers in kDa are shown to the right. Blot represents one from three independent experiments. Uncropped blots are presented in Original data. **B** Expression of RARα. SYO-1 (left panel) and MoJo (right panel) cells were treated with DMSO, 5 µM ATRA, 2.5 µM GSK343, or the combination of ATRA and GSK343 followed by staining with anti-RARα antibodies (green) or Phalloidin (red), respectively. Nuclei were visualized with DAPI (blue). One representative from three independent experiments is shown.

To assess effects of ATRA and GSK343 on RA signaling, we analyzed expression of proteins encoded by genes carrying RAREs [27]. The levels of the cyclin-dependent kinase (CDK) inhibitor p21 and the SRY-Box transcription factor 9 (SOX9) were increased after three days, while expression of the nuclear hormone receptor Peroxisome Proliferator Activated Receptor Gamma (PPARγ) and the proto-oncoprotein c-MYC was only attenuated after one week of combination treatment (Fig. 6A). As expected, mRNA levels of the four RA target genes *KLF4*, *NANOG, OCT4*, and *SOX2* [34] were altered. We found high expression of *NANOG* and *SOX2* in combination-treated cells, while *KLF4* and *OCT4* decreased following incubation with ATRA or GSK343, and did not further decline with the combination (Fig. S8).

**Fig. 7:**
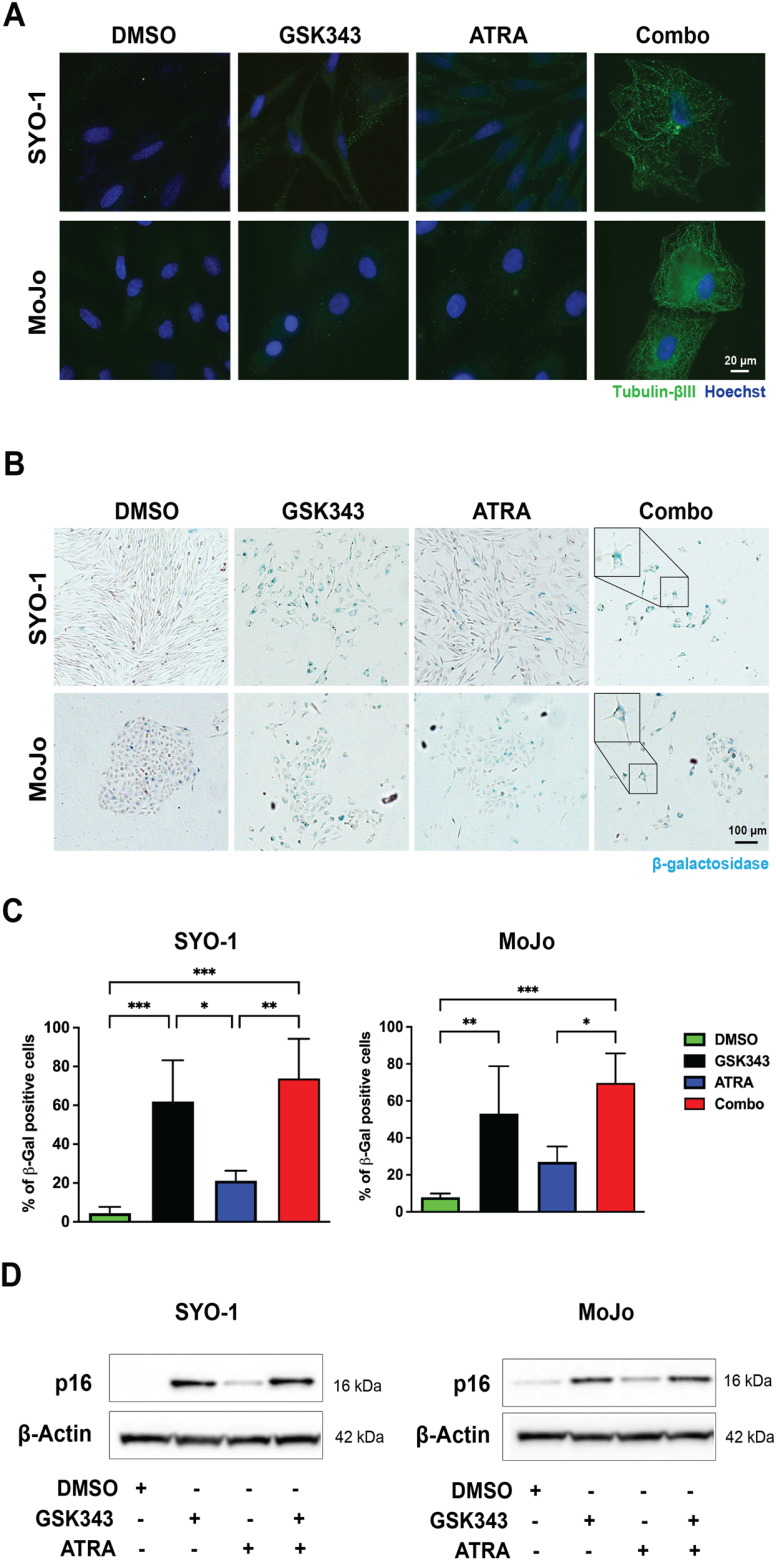
Effect of EZH2 inhibition and RAR activation on cellular senescence. **A** Expression of Tubulin-βIII. Immunofluorescence staining of Tubulin-βIII after inhibition of the RARα-PRAME-EZH2 ternary complex. SYO-1 and MoJo cells were treated for three weeks with DMSO, 2.5 µM GSK343, 5 µM ATRA individually or in combination (Combo), as indicated. Expression of Tubulin-βIII (green) and nuclei stained with Hoechst (blue). Representative images from three independent experiments are shown. **B** Assessment of senescence using β-gal staining. SYO-1 and MoJo cells were cultured on glass coverslips and treated with DMSO, 2.5 µM GSK343, 5 µM ATRA alone or in combination for ten days and stained for β-gal expression. **C** Quantification of the data shown in **B**. The graphs represent quantification of data obtained from at least four independent experiments. Statistical analysis: one-way ANOVA test with multiple comparisons. **D** Induction of p16 expression. SYO-1 and MoJo cells were treated with DMSO, 2.5 µM GSK343, 5 µM ATRA alone or in combination as indicated, and expression of the senescence marker p16 was analyzed using Western blot. β-Actin was used as a loading control. Molecular weight markers in kDa are shown to the right. One representative blot from three independent experiments is shown. Uncropped scans and quantifications are presented in Original data and Fig. S9B.

Noteworthy, the combination treatment affected the subcellular expression of RARα. Upon GSK343 exposure, RARα displayed a scattered pattern in the cytoplasm, while ATRA led to its accumulation in distinctive spots. Combined treatment resulted in perinuclear accumulation of RARα in MoJo cells (Fig. 6B). This effect was less pronounced in SYO-1 cells, likely due to their lower RARα expression compared to MoJo cells. Phalloidin staining showed changes in cell morphology, characterized by an enlarged cytoplasm with flattened actin filaments resembling the “fried egg” appearance of senescent cells [35]. To further analyze this observation, we assessed Tubulin-βIII protein levels and β-galactosidase (β-gal) activity and found that exposure to either GSK343 or the combined treatment indeed induced a strong expression of Tubulin-βIII as shown by immunofluorescence (Fig. 7A). In contrast, β-gal expressing cells were observed in all conditions, but the number of positive cells was larger in the GSK343 and combination-treatment groups (Fig. 7B, C). CellProfiler analysis revealed that SYO-1 cells became more circular and less elongated upon treatment, especially upon GSK343 exposure (Fig. S8A). The expression of the specific senescence marker p16 increased robustly with both GSK343 and GSK343 + ATRA in both cell lines (Figs. 7D and S9B), with a small but significant increase in p16 with the combination *versus* single treatment in SYO-1 cells. In summary, GSK343 or ATRA treatment affected RA targets and triggered senescence where EZH2 inhibition was dominant.

## Discussion

Synovial sarcoma (SS) is an aggressive STS with very limited therapeutic options [36] and with an urgent need for novel treatment modalities. Given the rarity of the disease, collecting homogeneous patient groups for epidemiological and molecular research is challenging, and cohort sizes are typically small. The characteristic SS18-SSX fusion protein has been regarded as a major driver in tumorigenesis [7, 8, 37], but its downstream effects are not fully uncovered. Here, we identified that SS18-SSX activates PRAME expression, which in turn disrupts the RA signaling pathway through interactions with RARα and EZH2 [18]. Our study revealed that targeting this ternary complex with an EZH2i or retinoic acid reduced proliferation and triggered senescence in SS.

By analysis of STS clinical samples and cell lines, we showed that high *PRAME* levels are specifically associated with SS. Our results demonstrated that PRAME upregulation is triggered by direct binding of SS18-SSX to the *PRAME* promoter, in line with a previous report on SS18 in ovarian cancer [38]. We further showed that SS18-SSX and PRAME expression are positively correlated both at mRNA and protein levels. Moreover, we found that *PRAME* is highly expressed in patients with recurrent/progressed disease, in accordance with a previous study linking *PRAME* to unfavorable survival in STS, although only 11% of cases were SS [39]. In contrast, *PRAME* levels were not significantly different between SS biological groups and had no impact on patient survival in the Chen cohort. This could be due to that all SS carry the SS18-SSX fusion protein, which induces *PRAME* expression, and any variances in levels might be too small to be a major prognostic factor. In addition, the cohort size may not be large enough to detect differences given that several factors influence the rate of survival and metastasis including but not limited to age, anatomical location, tumor size, surgical margins, or adjuvant treatment. Nevertheless, the overall expression of PRAME in SS makes it a promising target for treatment.

Further analysis of patient datasets revealed that the *PRAME* ^high^ patient group showed differentially expressed genes mainly involved in immune responses. This is in line with its first described role as a human antigen in melanoma that triggered an autologous cytotoxic T-cell immune response, thus a potential immunotherapy candidate [40, 41]. The process “RAR-bound genes” differed between the high and moderate *PRAME* groups, aligning with its described RA-repressor function via binding to EZH2 [18] which in turn leads to changes in transcription and chromatin remodeling by recruiting repressor complexes to target genes. Additionally, we identified deregulation in cell cycle and ribosome biogenesis pathways, consistent with its reported pro-tumorigenic functions [42, 43]. As for DNA repair and regulation of p53 activity, PRAME is known to be the receptor responsible for recognizing p14/ARF for degradation and allowing p53 induction upon DNA damage [44, 45]. Future research will clarify the molecular mechanisms behind the impact of PRAME on these key pathways, and how targeted therapies could be combined to treat SS patients.

Here we show that PRAME interacts with RARα and EZH2 in SS and hypothesized that single or simultaneous inhibition of EZH2 and/or ATRA treatment could restore RA signaling, which is repressed by the RARα-PRAME-EZH2 complex. Expression levels of the RA-targets c-MYC and PPARγ decreased after combined treatment with GSK343 and ATRA, resulting in the loss of pro-tumoral signals. As SS cells express stem cell-specific markers [46], we examined levels of *SOX2*, *OCT4*, *NANOG*, and *KLF4*, which have been reported to be RA responsive [47] as well as to participate in cellular reprogramming and homeostasis [48, 49]. Both *SOX2* and *NANOG* increased following combined ATRA and GSK343 treatment, in line with previous data showing higher SOX2 and NANOG levels upon RA exposure in embryonic stem cells [50]. SOX2 induction was also observed in induced pluripotent stem cells [51], and this gene has been shown to promote neural ectodermal differentiation [52]. We further found a reduction in *OCT4* which is consistent with previous reports following exposure to RA [53, 54]. While we also observed a drop in *KLF4*, ATRA has been shown to activate KLF4 expression in vascular smooth muscle cells, promoting stress fiber formation [55]. Importantly, RA is known to induce stemness or differentiation depending on the context [56].

The combination of GSK343 and ATRA was effective in reducing PRAME-EZH2 complexes, while in contrast to a previous study in melanoma [18], we did not detect any significant differences in PRAME-RARα complexes upon single ATRA treatment. This suggests that PRAME binds to RARα even in the absence of ligand in SS cells. Serendipitously we observed perinuclear distribution of RARα after combination treatment, especially in MoJo cells. This receptor translocates to the nucleus and activates target genes upon ligand binding followed by proteasomal degradation as a negative-feedback mechanism [57]. Besides its nuclear localization, RARα has also been found in neuronal dendrite RNA granules [58]. One possibility is that RARα might accumulate inside cellular organelles or vesicles, since we observed a scattered pattern in the cytoplasm and the perinuclear area following treatment, an observation that will need future investigation.

Previous studies showed that SS cells exhibited a mild response to retinoid treatment [59], and importantly RA is given to patients with neuroblastoma and acute promyelocytic leukemia [60, 61]. Our results showed that RA reduced cell proliferation but this might be insufficient for SS therapy due to high PRAME levels blocking signaling from the receptor. In contrast, single treatment with GSK343 robustly inhibited cell proliferation and colony formation, as previously reported for the EZH2i EPZ005687 in vitro [62] and tazemetostat both in vitro as well as in vivo in SS [63], outweighing the impact of ATRA when combined. This observation was supported by the results in the SS18-SSX and PRAME-negative SW982 cells, which barely responded to any of the treatments, and which had a higher IC_50_ for both compounds compared to SYO-1 TP-SS cells. In support, the SYO-1 *PRAME* knockdown cells were also unaffected by ATRA, thus demonstrating that the proposed therapeutic approach specifically targets PRAME-expressing SS. Previous studies have not reported synergy of EZH2is in combination with the standard of care chemotherapeutic agents including etoposide, topotecan, or doxorubicin in vitro [62]. While our data neither showed any synergistic effects of GSK343 and ATRA in SYO-1, a small synergy was observed in SW982 cells. Notably, the effects of EZH2 inhibition were more pronounced in SYO-1, even at the lowest concentration used, resulting in a 60% decrease in proliferation compared to only 12% in SW982 cells. This suggests that TP-SS cells are highly sensitive to GSK343, which likely overrides any putative synergy with ATRA. Importantly, long term inhibition of EZH2 might affect other biological processes unrelated to RA signaling. In the combination treatment, we found reduced c-MYC and increased p21 expression, which might rationalize the observed growth inhibition. While c-MYC is required for active proliferation of both normal and cancer cells, p21 is a potent cell cycle inhibitor. Notably, the *PRAME* knockdown cells also expressed high p21 levels, suggesting that reduced PRAME restores RA signaling.

Senescence is a cell fate characterized by stable arrest of cell proliferation, active metabolism, and the senescence-associated secretory phenotype (SASP) [35]. Here, we demonstrate that inhibition of EZH2 triggers this process in SS, with increased β-gal and p16 levels, together with elevated Tubulin-βIII and SOX9 expression following combination treatment. Tubulin-βIII is primarily expressed in neurons, but can be induced upon senescence in different tumor cells [64, 65] while the retinoid-inducible SOX9 protein [66] upregulates senescence factors, including p16 [67]. In contrast, it has been reported that apoptosis is induced upon EZH2i treatment in SS [63]. The metabolite, dosing, treatment duration, and cells used might explain this difference. Induction of senescence following EZH2 inhibition has previously been described in pancreatic adenocarcinoma resulting in activation of natural killer (NK) cells and T cell immunity [68, 69]. Future research will investigate the potential of combining this approach with PRAME-targeted immunotherapy in SS and other cancer types [40].

SS18-SSX alters the regulation of gene transcription by EZH2 via two processes: through its interaction with the Transducin-Like Enhancer Protein 1 (TLE1) [32], and as identified here, by direct induction of PRAME expression, which then recruits EZH2 to RA response promoter elements. This may in turn explain the dominant effect of EZH2 inhibition over RA signaling observed in some of the assays. The EZH2i tazemetostat has been used in a Phase II clinical trial (NCT02601950), showing a favorable safety profile in SS. While patients previously receiving several lines of treatments showed neither partial nor complete responses, the observation of stable disease in a subset shows the potential of further studying this approach, possibly in combination with other treatment options.

In conclusion, our data provide new mechanistic insights downstream of SS18-SSX. The direct upregulation of PRAME by the fusion protein leads to formation of a ternary complex with EZH2 and RARα, which results in repression of RA-induced anti-tumorigenic signaling. Furthermore, we demonstrate that combined EZH2 inhibition and ATRA treatment counteract SS cell proliferation by induction of cellular senescence (Graphical abstract). Similar to SWI/SNF-related matrix-associated actin-dependent regulator of chromatin subfamily B member 1 (SMARCB1) deficiency [63], we define high PRAME expression as a marker of sensitivity to EZH2 inhibition. While expressed at low levels only in certain normal tissues including testis, SS18-SSX drives high PRAME expression in SS. Importantly, our strategy could be a blue print for other RA-resistant cancers with high PRAME levels, including melanoma, breast, and non-small‐cell lung cancer [70].

## Supplementary information

Supplementary Information

## Data Availability

The datasets used and new data generated are cited in the main text and/or Supplementary Information. Uncropped Western blots and raw qPCR are found in Original data.
