# Supplementary material for: EZH2 inhibition sensitizes retinoic acid-driven senescence in synovial sarcoma
Source: Cell Death Dis. 2024 Nov 16;15(11):836. doi: 10.1038/s41419-024-07176-6 (PMC11569238; doi:10.1038/s41419-024-07176-6)
Supplement: Supplementary file 1 — Supplementary Information [file 41419_2024_7176_MOESM1_ESM.pdf]

Supplementary Information

EZH2 inhibition sensitizes retinoic acid-driven senescence in Synovial sarcoma

Muhammad Mushtaq[1,2,*,#], Judit Liaño-Pons[1,*,#], Jiansheng Wang[1], Mohammad Alzrigat[1], Ye Yuan[1], María Victoria Ruiz-Pérez[1], Yi Chen[3,4], Elena Kashuba[1,5], Felix Haglund de Flon[3], Bertha Brodin[6], and Marie Arsenian-Henriksson[1,#]

[1]Department of Microbiology, Tumor and Cell Biology (MTC), Biomedicum, Karolinska Institutet, SE-171 65, Stockholm, Sweden. [2]Present address: Department of Biotechnology, Faculty of Life Sciences and Informatics. Balochistan University of Information Technology, Engineering, and Management Sciences (BUITEMS), 87300, Quetta, Pakistan. [3]Department of Oncology-Pathology, Karolinska Institutet, Solna, 17176 Stockholm, Sweden. [4]Division of Hematology and Oncology, Department of Medicine, Columbia Stem Cell Initiative, Columbia University Irving Medical Center, New York, USA. [5]RE Kavetsky Institute of Experimental Pathology, Oncology and Radiobiology of NAS of Ukraine, 03022 Kyiv, Ukraine. [6]Department of Applied Physics, Biomedical and X-Ray Physics, KTH Royal Institute of Technology, SE-10691 Stockholm, Sweden.

* Authors contributed equally

# Corresponding authors:

muhammad.mushtaq1@buitms.edu.pk

judit.liano.pons@ki.se

marie.arsenian.henriksson@ki.se

Table of contents

Supplementary Material and Methods

Figures S1-S9

Tables S1-S4

Fig. S1. Effects of *PRAME* expression for SS patient survival and levels of PRAME and EZH2 proteins in soft tissue sarcoma (STS) cells. The Figure corresponds to main Figure 1.

Fig. S2. Gene set enrichment analysis (GSEA) of *PRAME* ^{high} *versus PRAME* ^{moderate}. The Figure is related to main Figure 2 and additional data is presented in Table S1.

Fig. S3. KEGG pathway analysis. KEGG enrichment analysis of the DEGs presented in main Figure 2A-B.

Fig. S4. Effect of GSK343 and ATRA on the RARα-PRAME-EZH2 ternary complex. Data related to main Figure 4.

Fig. S5. Viability and colony formation assays in SS cells after treatment with GSK343 and ATRA. Figure corresponds to main Figure 5.

Fig. S6. Combinatorial treatments of GSK343 and ATRA in SS cells. Figure corresponds to main Figure 5.

Fig. S7. PRAME and p21 protein levels in *shControl* and *shPRAME* cell lines. Figure related to main Figure 5.

Fig. S8. Effects on RA target genes after GSK343 and ATRA treatment. Figure corresponds to main Figure 6.

Fig. S9. Senescence parameters after treatment in SS cells. Figure is related to main Figure 7.

Table S1. Detailed enrichment results of the gene sets. Data corresponds to the GSEA graphs shown in main Figure 2 and Figure S3.

Table S2. List of cell lines employed.

Table S3. List of Antibodies used in Western blot assays.

Table S4. List of Antibodies used in immunofluorescence (IF) and Proximity Ligation Assays (PLAs).

Supplementary References

Supplementary Materials and Methods

Cell Culture

SS18-SSX translocation positive SS cells, SYO-1, HSSY-II, and MoJo, translocation-negative SS (SW982) [1,2], rhabdomyosarcoma (RH-30), liposarcoma (SW872), Ewing sarcoma (RD-ES), and leiomyosarcoma (SK-UT-1) cell lines were used (Table S2). The SS and RD-ES cell lines were cultured in DMEM, RH30 and SW872 in RPMI, while SK-UT-1 cells in MEM. All cell lines were maintained in 10% fetal bovine serum (FBS) (Life Technologies, Waltham, MA, USA), except MoJo cells, which were cultured in 15% FBS. All media were supplemented with 100 units/ml penicillin and 100 µg/ml streptomycin (Merck/Sigma-Aldrich, St. Louis, MO, USA). Cells were cultured at +37°C in humified conditions with 5% CO_2, authenticated [3], and tested negative for mycoplasma (MycoAlert Mycoplasma Detection Kit, Lonza, Basel, Switzerland).

The following treatments were employed throughout the study: 5 µM ATRA and 2.5 µM GSK343, individually or in combination, with DMSO as vehicle. All compounds were purchased from Merck/Sigma-Aldrich (St. Louis, MO, USA).

Lentiviral Transduction for PRAME Knockdown

Knockdown of PRAME was carried out by lentiviral transduction in SYO-cells. Lentiviral particles carrying the pLKO.1 puro non-mammalian shRNA control vector (SHC002V) or shRNAs against human *PRAME*, sh*PRAME1* (TRCN0000296271), and sh*PRAME2* (TRCN0000115704) were purchased from Merck/Sigma-Aldrich (St. Louis, MO, USA). SYO-1 cells were seeded at a confluence of 25,000 cells/well in 6-well plates the day before transduction. Complete medium was then replaced by 1 ml of medium without FBS containing lentiviral particles at MOI = 3 with 4 µg/ml of polybrene (VectorBuilder GmbH, Neu-Isenburg, Germany) to increase efficiency. After 12 h, 1 ml of complete medium was added to each well, which was refreshed after 24 h. Forty-eight hours post-transduction, cells were selected with 1 µg/ml puromycin (Merck/Sigma-Aldrich, St. Louis, MO, USA) for two days and then maintained with 0.5 µg/ml puromycin.

mRNA Expression Analysis of *PRAME* in STS

The R2 Genomic Platform (http://r2platform.com) was used to study the expression pattern of *PRAME* in STS. We combined data from the Boshoff [4] and the Filion [5] cohorts for analysis of *PRAME* expression in a panel of STS clinical samples. Briefly, SS (n=56),

rhabdomyosarcoma (n=30), Ewing Sarcoma (n=33), and leiomyosarcoma (n=8) patient data were examined. Log2 values of RNA expression levels were extracted and subjected to statistical analysis using GraphPad Prism 6 software (La Jolla, CA, USA). One-way ANOVA test was applied for statistical analysis. We also analyzed the mutational status of *PRAME* in SS tumors using the COSMIC database [6]. The query was generated as follows: (https://cancer.sanger.ac.uk/cosmic/browse/tissue?wgs=off&sn=soft_tissue&ss=all&hn=synovial_sarcoma&sh=all&in=t&src=tissue&all_data=n#genes, accessed 14/05/2024).

Synovial sarcoma patient data from the adult soft tissue sarcomas TCGA cohort (n=10) was analyzed using cBioportal [7–9]. The mRNA levels of *PRAME* (in RNASeq V2 RSEM normalized values) were correlated with "disease free status" and "overall survival status" and analyzed with unpaired *t*-test for significance.

Additionally, we leveraged the Karolinska Hospital SS (Chen) cohort, which encompasses RNA-seq expression profiling along with patients clinical metadata [10]. The gene expression data was quantified and presented in two formats: raw counts and transcripts per million (TPM). For subsequent analytical procedures, TPM values were log-transformed using the formula log2(TPM + 1). Variables that deviated from non-Gaussian distribution were subjected to the Mann-Whitney U test for statistical evaluation. Survival analysis were conducted utilizing the Kaplan-Meier estimator, as implemented in the "survminer" and "survival" R packages, with the log-rank test employed to facilitate comparative analyses between distinct cohorts.

mRNA expression analysis of *PRAME*, *EZH2*, and *RAR genes* in STS cell lines were analyzed through mining the publicly available data from the Cell Line Encyclopedia (Depmap portal). The Logp1 values of mRNA expression were downloaded and applied to GraphPad Prism (GraphPad Software 9.0, La Jolla, CA, USA) for statistical analysis (one-way ANOVA with multiple comparison test).

In silico Gene Expression Analysis

Gene expression analysis in tumor samples was performed using Microarray expression data from the GSE40018 and GSE40021 datasets. GSE40018 included 34 SS samples from the GPL13497 platform (Agilent-026652 Whole Human Genome Microarray 4 × 44K v2). The GSE40021 dataset contained 58 SS samples from the GPL6480 platform (Agilent-014850 Whole Human Genome Microarray 4 × 44K G4112F). The information from these two data sets was combined for analysis using the Robuse Multi-Array Average algorithm and combat function of the "sva" package in R (v4.1.3) for preprocessing and removal of batch effects.

Noteworthy, previous studies have shown that all SS samples expressed *PRAME* at certain levels [11], hence we grouped the samples only into *PRAME* moderate and high groups. The median *PRAME* expression was used as the cutoff value to make the division into the high and moderate *PRAME* expressing groups, respectively.

Differential Gene Expression and Gene Set Enrichment Analysis

Differentially expressed genes (DEGs) were analyzed using the limma package in R [12]. Gene ontology (GO) and Kyoto Encyclopedia of Genes and Genomes (KEGG) enrichment analyses were performed using clusterProfiler packages in R [13] to identify the pathways in which the DEGs are involved. To analyze the involvement of *PRAME* in RA signaling in SS, the RA-target gene set "DELACROIX_RAR_BOUND_ES.gmt" was downloaded from the Molecular Signatures Database (MSigDB) [14] and gene set enrichment analysis (GSEA) was performed using the GSEA software (Broad Institute, v4.2.3) (Table S1). The molecular profile data of *PRAME* expression in SS as described above was used for GSEA.

Knockdown of SS18-SSX

The SYO-1 and MoJo cells were cultured in 6 cm dishes. When the cultures were 90-95% confluent, cells were transfected with control siRNA or siRNA specific for either *SSX1* (MoJo) or *SSX2* (SYO-1). Lipofectamine 3000 transfection reagent (Cat No. L3000008, Invitrogen, CA, USA) was used for transfection, following the manufacturer's protocol. The sequences of siRNA pools from GE Healthcare Dharmacon (Lafayette CO, USA) can be found below. Cells were harvested 48 hours post transfection.

siRNAs used for SS18-SSX Knockdown

ON-TARGETplus Human SSX2 (6757) siRNA - SMARTpool, L-017245-01-0005

5′-UUGAUGAUAUUGCCACAUA-3′

5′-CAGCAGAGGACGAAAAUGA-3′,

5′-CCUUAUAAUUGAUGAGCAA-3′,

5′-ACAGAAUCAUCCCGAAGAU-3′

ON-TARGETplus Human SSX1 (6756) siRNA - SMARTpool, L-019194-00-0005

5′-UCUGCAUGGAACAGCAUUA-3′,

5′-AAGAGAAAGUAUGAGGCUA-3′,

5′-GUUCGAUGUUAGCGUUUAC-3′,

5′-ACUGUGUCAUUCUGUUAGA-3′

ON-TARGETplus Non-targeting Pool

5′-UGGUUUACAUGUCGACUAA-3′,

5′-UGGUUUACAUGUUGUGUGA-3′,

5′-UGGUUUACAUGUUUUCUGA-3′,

5′-UGGUUUACAUGUUUUCCUA-3′

SS18-SSX Expression Construct and Cell Transfection

The cDNA of *SS18-SSX1* was cloned in the eukaryotic expression vector pTARGET (Promega, Madison, Wis, USA) as previously described [14]. The SW982 cells were transfected using Lipofectamine 3000 transfection reagent (Cat No. L3000008, Invitrogen, Thermo Fisher Scientific, Waltham, MA, USA). Cells were harvested after three and ten days for expression analysis at mRNA and protein, respectively.

Chromatin Immunoprecipitation Assay

The EZ-Magna ChIP™ G-Chromatin Immunoprecipitation kit (Cat No. 17-409) was purchased from EMD Millipore (Burlington, MA, USA). Briefly, SYO-1 and MoJo cells were grown in 10 cm culture dishes. When the culture was 90% confluent, cells were fixed at room temperature for 10 min in a medium containing 1% methanol-free formaldehyde (Cat No. 28908, Thermo Fisher Scientific, Waltham, MA, USA) and then the manufacturer's protocol was followed. The extracted nuclei were sheared using 20 cycles of Misonix Ultrasonic Liquid Processor (S-4000) set to 30 s "on" and 30 s "off" per cycle with an amplitude of 50%. For each ChIP reaction, 120 µg of sheared DNA was used. After overnight incubation with an anti-SS18-SSX antibody (Cat No. 70929, Cell Signaling Technology, Leiden, Netherlands) or an isotype control IgG antibody (rabbit IgG, polyclonal ab171870; Abcam, Cambridge, UK), protein G beads (EMD Millipore, Burlington, MA, USA), and the DNA-protein complex, reverse cross linking was performed using a Thermomixer (Invitrogen, Thermo Fisher Scientific, Waltham, MA, USA) at +67°C for 2 hours, then 4 µL of 20 mg/mL proteinase K

(MD Millipore, Burlington, MA, USA) were added and the mixture was incubated at +57°C for another 2 hours.

Quantitative PCR was performed on Step One Real-Time PCR Systems (Applied Biosystems, Van Allen Way, CA, USA). Nucleotides -195 to -97 of the *EGR1* promoter were amplified as a positive control while the region spanning -1015 to -823 bp upstream of the *EGR1* transcription start site was used as a negative control. The -66 to +35 bp sequence in relation to the *PRAME* transcription start site was used for analysis.

The fold enrichment method was applied to detect the binding of SS18-SSX to the promoter regions of the *PRAME* or *EGR1* genes. Unpaired *t*-test with Welch's correction was applied to calculate the *p*-values of at least three independent experiments using GraphPad Prism 6.0 software (La Jolla, CA, USA). Raw values can be found in Original data.
Sequences of primers used for ChIP-qPCR:

$EGR1^{-195/-97}$ _Forward: *5´*-TAGGGTGCAGGATGGAGGT-*3´*
$EGR1^{-195/-97}$ _Reverse: *5´* -AAGCAGGAAGCCCTAATATGGCAG-*3´*
$PRAME^{-66/+35}$ _Forward*:* *5´*-GGGGAGCTGTACCCTGAAG-*3´*
$PRAME^{-66/+35}$ _Reverse: *5´*-CTGGGAGGAAGTGGGTTTT-*3´*
$EGR1^{-1015/-823}$ _Forward: *5´*-AATCAGCTTCCCCACTTCGG-*3´*
$EGR1^{-1015/-823}$ _Reverse: *5´*-GGATAGGAAGTCACGGCCAC-*3´*

Cell Viability Assay

For 3-(4,5-dimethylthiazol-2-yl)-2,5-diphenyltetrazolium bromide (MTT) based cell viability assay, 1,500 SYO-1, MoJo, or HSSY-II cells were seeded per well in 96 well plates and cultured overnight. Next day, the cells were exposed to 2.5 µM GSK343, 5 µM ATRA, or in combination using DMSO as a vehicle.
Briefly, at the end of each treatment period, 0.5 mg/mL of MTT (Merck/Sigma-Aldrich, St. Louis, MO, USA) was added to each well and incubated at +37°C for 1 hour. Medium was then replaced with DMSO followed by incubation on a shaker at room temperature for 30 min. The OD was measured at 570 nm using a LUMIstar Omega plate reader (BMG Labtech, Ortenberg, Germany). Three independent experiments with each cell line were performed and multiple regression was used for statistical analysis.

Cell Counting Assay

Cells were seeded in 6-well plates and incubated for either three or seven days with DMSO, 5

μM ATRA, 2.5 μM GSK343, or the combination of ATRA and GSK343. For the seven days assay, one well was pre-treated with 2.5 μM GSK343 for 72 h then switched to combination treatment until day seven. Cell lines were seeded at 50,000 cells/well for both time points except for SYO-1 cell line, which was seeded at 25,000 cells/well for the seven-day treatment. At each time point, cells were washed with 1X PBS, trypsinized, and resuspended in 1 ml complete growth medium. Cells were counted using the Muse® Cell Analyzer (MerckMillipore, St. Louis, MO, USA).

BrdU Cell Proliferation Assay

SYO-1 and SW982 cells were seeded in 96 well plates and treated with DMSO, 5 μM ATRA, 2.5 μM GSK343, or the combination of ATRA and GSK343 for seven days. Proliferation was measured using CyQUANT™ Cell Proliferation Assay kit (C35006, Thermo Fisher Scientific, Waltham, MA, USA), according to the manufacturer's instructions. After 1 h of incubation, fluorescence was measured using a SpectraMax i3x reader (Molecular Devices, Sunnyvale, CA, USA) with SoftMax Pro version 7.1 software. Three independent experiments were used to determine the half maximal inhibitory concentration (IC_{50}) values, which were determined by a nonlinear regression analysis using GraphPad Prism 9 software (La Jolla, CA, USA).

Synergy assessment

SYO-1 and SW982 cells were seeded in 96-well plates and treated with different concentrations of GSK343, ATRA, or the combinations for 72 h and seven days. Cell proliferation was determined by BrdU assay and normalized to control. Data was transferred to SynergyFinder [15], and the Bliss synergy score was calculated. At least three independent experiments were performed for each cell line.

IncuCyte Live-Cell Proliferation Imaging

The sh*Control*, sh*PRAME1,* and sh*PRAME2* SYO-1 cells were seeded in 96-well plates in triplicates and treated for seven days with ATRA or DMSO. Medium plus ATRA or DMSO was changed once in the middle of the treatment period. Cell proliferation was assessed by measuring confluence using the Incucyte® S3 Live-Cell imaging system (Sartorius, Goettingen, Germany). Four bright-field images per well were taken every six hours. Confluence was normalized versus the first time-point (0 h) and presented as the mean of three

independent experiments. Statistical significance was determined with multiple unpaired *t*-tests.

Gene Expression Analysis by RT-qPCR

Total RNA was extracted using RNeasy Plus Mini Kit (Qiagen, Germantown, MD, USA) following the manufacturer's protocol. RNA was used to synthesize cDNA using First Strand Synthesis Kit (Thermo Fisher Scientific, Waltham, MA, USA) following the manufacturer's protocol. RT-qPCR was performed using StepOne™ Real-Time PCR System (Applied Biosystems, Van Allen Way, CA, USA). The primer concentration was adjusted to a final concentration of 3 µM and the total reaction volume in all RT-qPCR experiments was 20 µL, using a SYBR Green master mix (Thermo Fisher Scientific, Waltham, MA, USA). The primers sequences (5′ to 3′) were as follows.

*KLF4*_Forward: *5′*-CCCACATGAAGCGACTTCCC-*3′*
*KLF4*_Reverse: *5′*-CAGGTCCAGGAGATCGTTGAA-*3′*
*OCT4*_Forward: *5′*-CTGGGTTGATCCTCGGACCT-*3′*
*OCT4*_Reverse: *5′*-CCATCGGAGTTGCTCTCCA-3′
*SOX2*_Forward: *5′*-GCCGAGTGGAAACTTTTGTC-*3′*
*SOX2*_Reverse: 5′-GGCAGCGTGTACTTATCCTTCT-*3′*
*NANOG*_Forward: *5′*-TTTGTGGGCCTGAAGAAAACT-*3′*
*NANOG*_Reverse: *5′*-AGGGCTGTCCTGAATAAGCAG-*3′*
*TBP*_Forward: *5′*-GAGAGTTCTGGGATTGTACCG-*3′*
*TBP*_Reverse: *5′*-ATCCTCATGATTACCGCAGC-*3′*
*β–Actin*_Forward: *5′*-CACCATTGGCAATGAGCGGTTC-*3′*
*β–Actin*_Reverse: *5′*-AGGTCTTT GCGGATGTCCACGT-*3′*
*GAPDH*_Forward: *5′*-ACCACCCTGTTGCTGTAGCCAA-*3′*
*GAPDH*_Reverse: *5′*-GTCTCCTCT GACTTCAACAGCG-*3′*

Relative gene expression was normalized to that of the transcript from the genes encoding the *TATA-binding protein* (*TBP*), *GAPDH,* and *β–Actin*, which served as endogenous controls. All experiments were performed independently at least three times and two-way ANOVA analysis was performed for statistical analysis. Raw values can be found in Original data.

Proximity Ligation Assay

SYO-1 and MoJo cells were seeded in six well plates containing glass coverslips. The following

day, they were treated with DMSO, 5 µM ATRA, 2.5 µM GSK343, or the combination of ATRA and GSK343 for three days. Cells were washed twice with PBS, fixed with 4% formaldehyde, and permeabilized with 0.1% Triton X-100. Next, slides were blocked with blocking buffer and incubated overnight at +4°C with primary antibodies against PRAME together either with a RARα or an EZH2 antibody (1:200) (Table S4). Proximity ligation was performed utilizing the Duolink® *In Situ* Green Starter Kit Mouse/Rabbit (Merck/Sigma-Aldrich (St. Louis, MO, USA) according to the manufacturer's protocol. An additional staining with Phalloidin Dylight TM 554 (1:800, 13054S, Cell Signaling, Danvers, MA, USA) was performed for 45 min at RT. Co-localization fluorescence signal intensity was detected using a Zeiss Axiovert 200 M microscope with the Zen 2 blue edition software and Z-stack pictures with a confocal microscope Zeiss LSM700 (Oberkochen, Germany). Three independent experiments were performed, signal intensity was quantified using Image J (NIH, Bethesda, MD, USA), and represented as relative to the number of nuclei. One-way ANOVA with multiple comparisons test was used for statistical analysis.

Senescence Detection Assay

10,000 cells/well for the control and 20,000 cells/well for the treatments were seeded and incubated for ten days with DMSO, 5 µM ATRA, 2.5 µM GSK343, or the combination of ATRA and GSK343. The proportion of senescent cells was measured using the Senescence β-galactosidase (β-gal) staining Assay Kit (Millipore KAA002, Burlington, MA, USA). For quantification, the number of β-gal positive cells was counted in each culture under the microscope and representative images were captured. The assay was performed at least four independent times and after quantification of β-gal staining, one-way ANOVA with multiple comparisons test was applied for statistical significance.

Quantification of Morphological Features of Scenescent Cells

Images of SYO-1 cell line from the β-gal senescence assay were analyzed using CellProfiler 4.2.6 software (Broad Institute of MIT and Harvard, Cambridge, MA, USA) to calculate Form Factor, Eccentricity, and Max Feret Diameter parameters [16]. FormFactor equal to 1 represents a perfectly circular cell, while 0 represents an irregular shape. Eccentricity is the ratio of the distance between the foci of the cell and its major axis length. A cell whose eccentricity equals 0 is a circle, while a cell whose eccentricity is 1 is a line segment. Max Feret Diameter measures

the largest possible diameter between the two most distant points in a cell. One-way ANOVA with multiple comparison test was used for statistical significance.

Colony Formation Assay

200 cells were seeded in each well of a 6 well plate and treated after 24 h with DMSO, 5 µM ATRA, 2.5 µM GSK343, or the combination of ATRA and GSK343 at +37°C. Treatments were maintained for two weeks for SYO-1 and four weeks for MoJo cells, with medium plus compounds being changed every three days. Cells were fixed in 4% paraformaldehyde (Merck/Sigma-Aldrich, St. Louis, MO, USA) and stained with 0.2% crystal violet solution (Merck/Sigma-Aldrich, St. Louis, MO, USA), followed by several washes with water to remove excess of dye. Images were taken using a ChemiDoc XRS+ System (Bio-Rad, Hercules, CA, USA). Experiments were repeated at least three times for each cell line and one-way ANOVA with multiple comparisons test was performed for statistical analysis.

Western Blot Analysis

Western blot analysis was performed as described [17]. Briefly, whole-cell lysates were prepared using RIPA lysis buffer containing protease inhibitor cocktail (Roche, Basel, Switzerland). Samples were boiled in 1× Laemmli buffer at +95°C for 10 min and separated by sodium dodecyl sulfate-polyacrylamide gel electrophoresis (SDS-PAGE) (BoltTM 4-12% Bis-Tris Plus, Invitrogen, CA, USA). After gel electrophoresis, separated proteins were transferred to nitrocellulose membranes (Trans-Blot Turbo Transfer Pack, Cat No. 1704159, Bio-Rad, Hercules, CA, USA). Membranes were blocked with 5% non-fat milk (AppliChem GmbH, Darmstadt, Germany) and probed with primary antibodies overnight at +4°C. Horseradish peroxidase tagged anti-mouse or anti-rabbit secondary antibodies (dilution 1:3000; Agilent Technologies, Santa Clara, CA, USA) were added followed by incubation for 1 h and development using SuperSignal™ West Dura (Cat No. 34076, Thermo Fisher Scientific, Waltham, MA, USA). Immunoblots were imaged using a ChemiDoc XRS+ System (Bio-Rad, Hercules, CA, USA) and analyzed with Image Lab software (Bio-Rad, Hercules, CA, USA). GAPDH or β-Actin antibodies were used as loading controls. The PageRuler™ Plus Prestained protein ladder was used (Cat No. 26620, Thermo Fisher Scientific, Waltham, MA, USA). All Western blot experiments were performed at least three times. The used antibodies can be found in Table S3 and the uncropped Western blots in Original data.

Immunofluorescence Analysis

Synovial sarcoma cells were treated with DMSO, ATRA, GSK343, or the combination of ATRA and GSK343, for ten days. Immunofluorescence staining was performed for analysis of Tubulin-βIII and RARα. Two different protocols were used.

Synovial sarcoma cells (3,000) were cultured per well of six well plates containing glass coverslips. Next day the culture was treated with DMSO, 5 μM ATRA, 2.5 μM GSK343, or the combination of ATRA and GSK343, and incubated at +37°C for ten days. Cells were fixed in a 1:1 solution of methanol and acetone and stored at -20°C. Cells on coverslips were rehydrated for 30 min in PBS and incubated with mouse anti-Tubulin-βIII antibody (1:2000) for 1 hour at room temperature. After three washes with PBS, the coverslips were incubated with rabbit anti-mouse FITC-conjugated secondary antibody (1:700; Invitrogen, Waltham, MA, USA) and Hoechst (Merck/Sigma-Aldrich, St. Louis, MO, USA) was added to stain cell nuclei.

For RARα immunofluorescence, treated SYO-1 and MoJo cells were fixed with 4% paraformaldehyde solution (Merck/Sigma-Aldrich, St. Louis, MO, USA) for 15 min and incubated with blocking solution (3% BSA and 0.25% Triton X-100 in PBS) for 1 h at room temperature. Coverslips were incubated with anti-RARα in blocking solution (1:250, PA1-810A, Thermo Fisher Scientific, Waltham, MA, USA) overnight at +4ºC. Next day, samples were washed three times with PBS and incubated with AlexaFluor 488-conjugated goat anti-rabbit secondary antibody (1:700, Invitrogen, Waltham, MA, USA), and Phalloidin Dylight TM 554 (1:800, 13054S, Cell Signaling, Danvers, MA, USA) for 45 min at room temperature. After three washes with PBS, DAPI (1:10,000; Merck/Sigma-Aldrich, St. Louis, MO, USA) was added to stain nuclei. Following 5 min incubation, coverslips were washed three times in PBS and mounted on slides with ProLong™ Diamond Antifade Mountant (P36961, Thermo Fisher Scientific, Waltham, MA, USA). Images were captured with a Zeiss Axiovert 200M microscope with the Zen 2 blue edition software and edited using ImageJ (NIH, Bethesda, MD, USA). All immunofluorescence experiments were independently performed three times with each cell line. The used antibodies can be found in Table S4.

Statistical Analysis

All data are represented as mean ± standard deviation (SD) of at least three independent biological experiments. No statistical methods were used to predetermine sample size. Statistical tests are specified for every method and in Figure legends, and calculated using

GraphPad Prism (GraphPad Software 9.0, La Jolla, CA, USA). For unpaired t-test, two-sided test was conducted, and Tukey's multiple comparison tests were performed for one-way and two-way ANOVA. Significance is highlighted with *, **, ***, and **** indicating p-value < 0.05, p-value < 0.01, p-value < 0.001, and $p < 0.0001$, respectively, and "ns" as non-significant.

Supplementary Figures

A

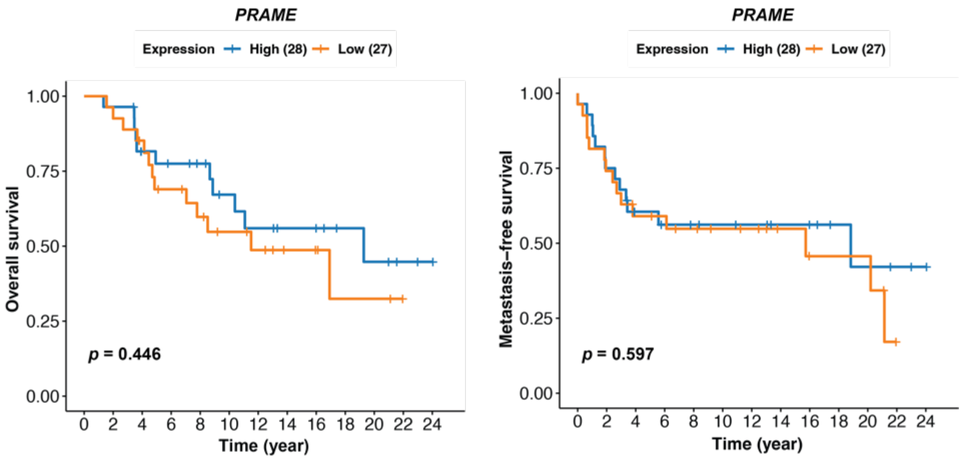

B

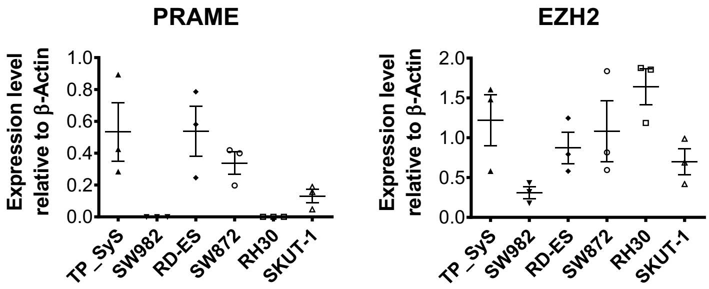

Fig. S1. Effects of *PRAME* expression for SS patient survival and levels of PRAME and EZH2 proteins in soft tissue sarcoma (STS) cells. (A) Overall (left panel) and metastasis-free survival (right panel) of SS patients from the Chen cohort divided in High *versus* Low *PRAME* expression. Statistical analysis: log-rank test. **(B)** The expression level of PRAME and EZH2 relative to expression of β-Actin was quantified and presented in graphs. Notably, TP-SS represents expression of PRAME and EZH2 in all three SS18-SSX-translocation positive SS cells (SYO-1, MoJo, and HSSY-II) while TN-SS refers to the translocation negative SS SW982 cell line. The Figure corresponds to main Figure 1E.

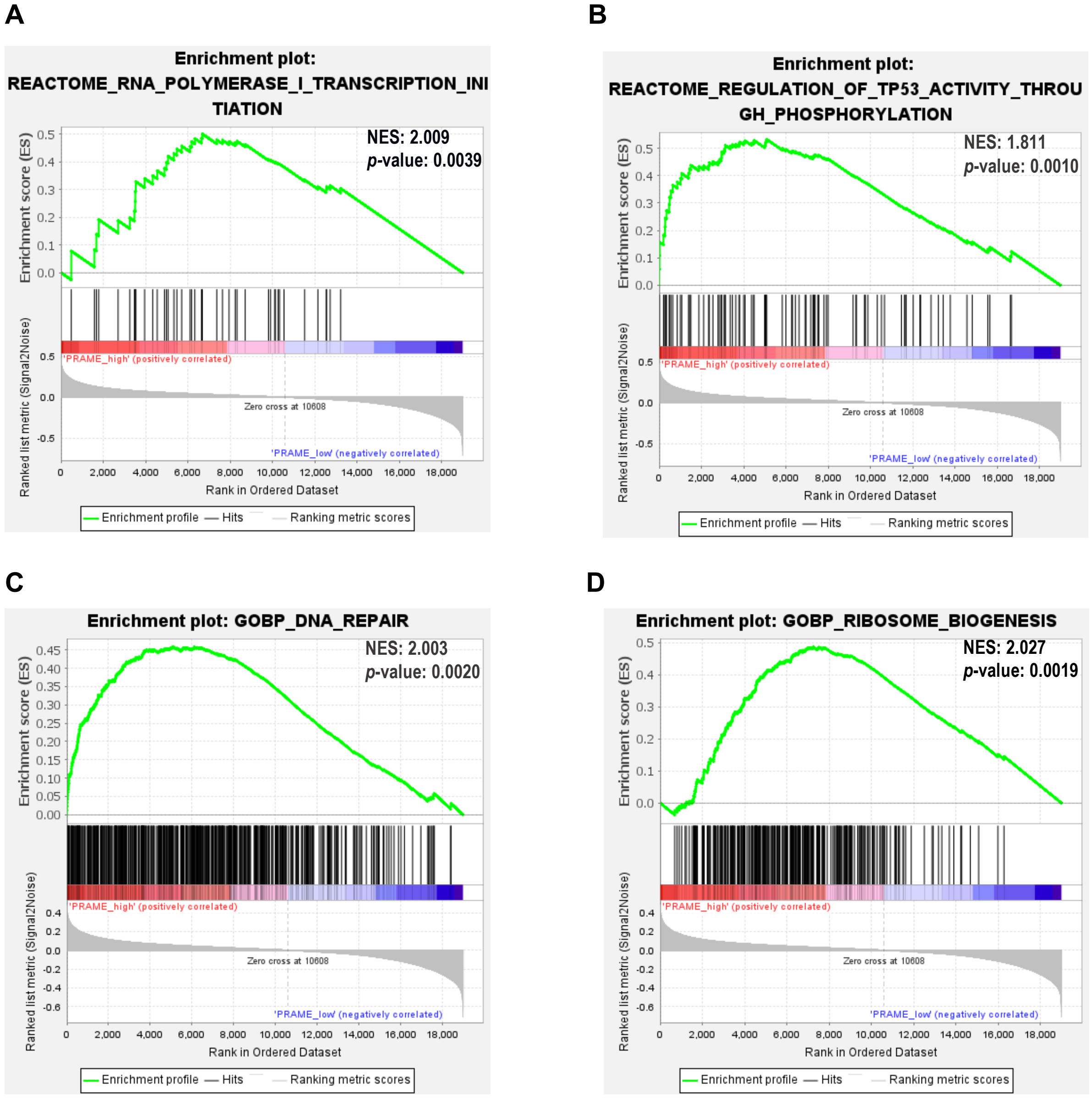

Fig. S2. Gene set enrichment analysis (GSEA) of *PRAME* high *versus PRAME* moderate. Gene set enrichment analysis (GSEA) was performed for **(A)** RNA Polymerase transcription initiation transcriptome, **(B)** Regulation of TP53 activity through phosphorylation **(C)** DNA repair, and **(D)** Ribosome biogenesis, using gene sets in the GSEA Molecular Signatures Database. "Signal-to-Noise" ratio (SNR) statistics was used to rank the genes per their correlation with *PRAME* high (red) or *PRAME* moderate (blue) expression. The green curves correspond to the enrichment score (ES) curve, which is the running sum of the weighted enrichment score obtained from GSEA software. The normalized enrichment score (NES) and the *p*-values for each gene set are shown in the graphs. The Figure is related to main Figure 2 and additional data is presented in Table S1.

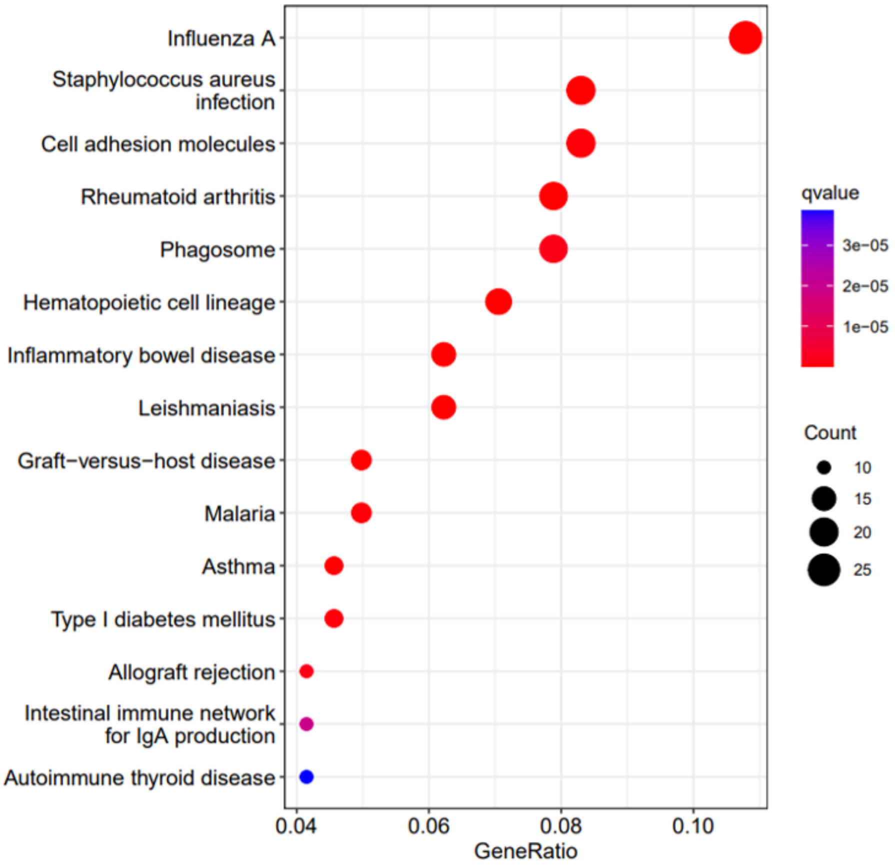

Fig. S3. KEGG pathway analysis. KEGG enrichment analysis of the DEGs presented in Figure 2A-B. The y-axis shows clustered KEGG pathways. The GeneRatio on the x-axis represents the ratio of the number of genes enriched in one KEGG pathway to the number of upregulated or downregulated DEGs. The indicator bar indicates significance as *q*-value and the black circles represent number of counts. The Figure is related to main Figure 2.

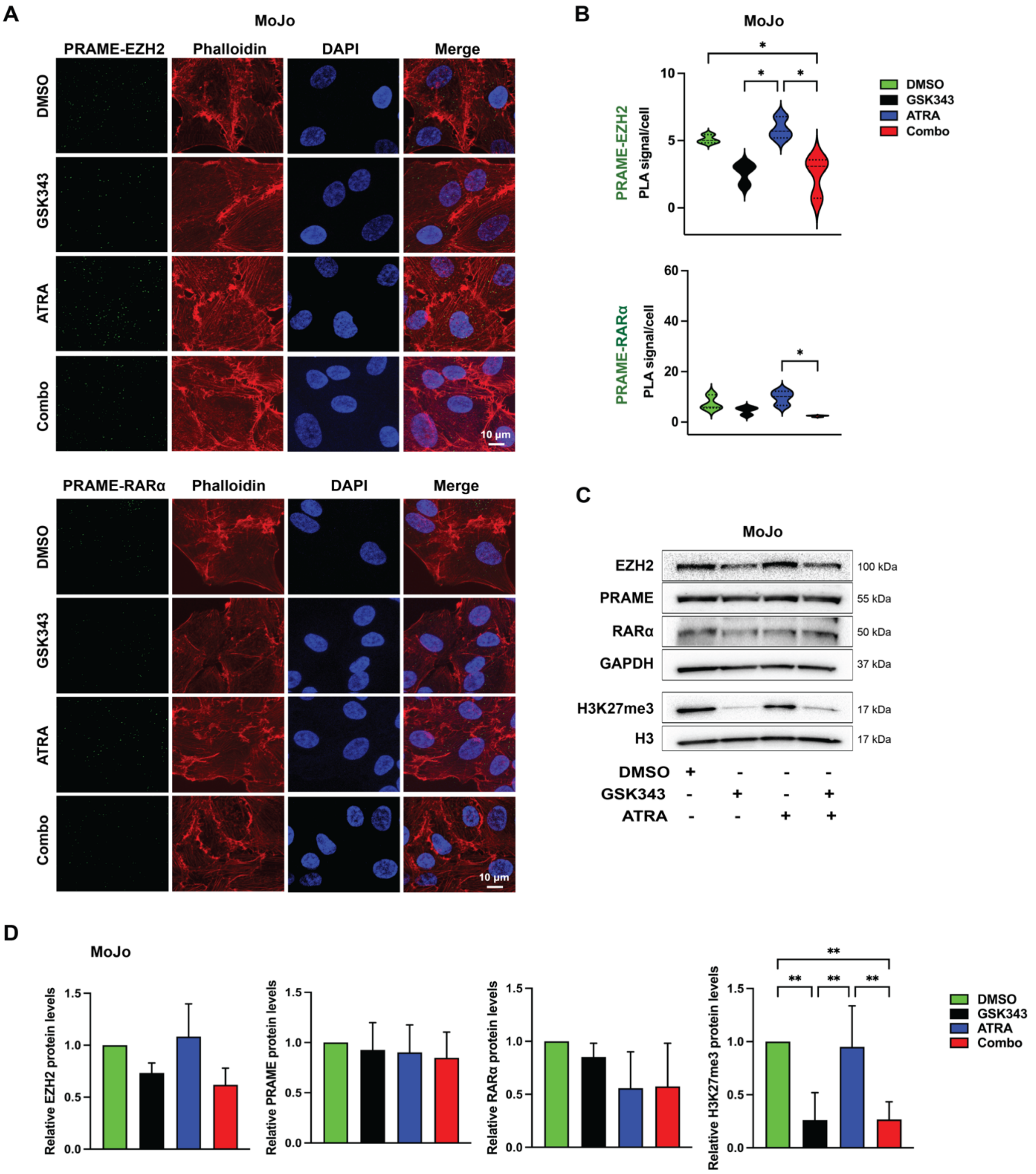

Fig. S4. Effect of GSK343 and ATRA on the RARα-PRAME-EZH2 ternary complex. (A) Proximity ligation assay (PLA)-based analysis of PRAME-EZH2 colocalization (upper panel) and PRAME-RARα (lower panel) in MoJo cells treated with DMSO, 2.5 μM GSK343, 5 μM ATRA individually or in combination for 72 h. The PLA signal is shown in green, and nuclei were stained with DAPI (blue). **(B)** Quantification of the PLA signal/cell. The violin plots represent data from at least three independent experiments. For statistical analysis one-way ANOVA with multiple comparison tests was performed. **(C)** Protein expression by Western blot of EZH2, PRAME, RARα, and H3K27me3 in MoJo after 72 h treatment. GAPDH was used as a loading control, and total H3 for H3K27me3. Molecular weight markers in kDa are shown to the right. Representative blots out of three independent experiments are shown, and images of the uncropped scans are presented in Original data. **(D)** Quantification of Western blot in (C). Protein levels were normalized against the loading control and represented as fold change of the control (DMSO). For statistical analysis one-way ANOVA with multiple comparison tests was performed.

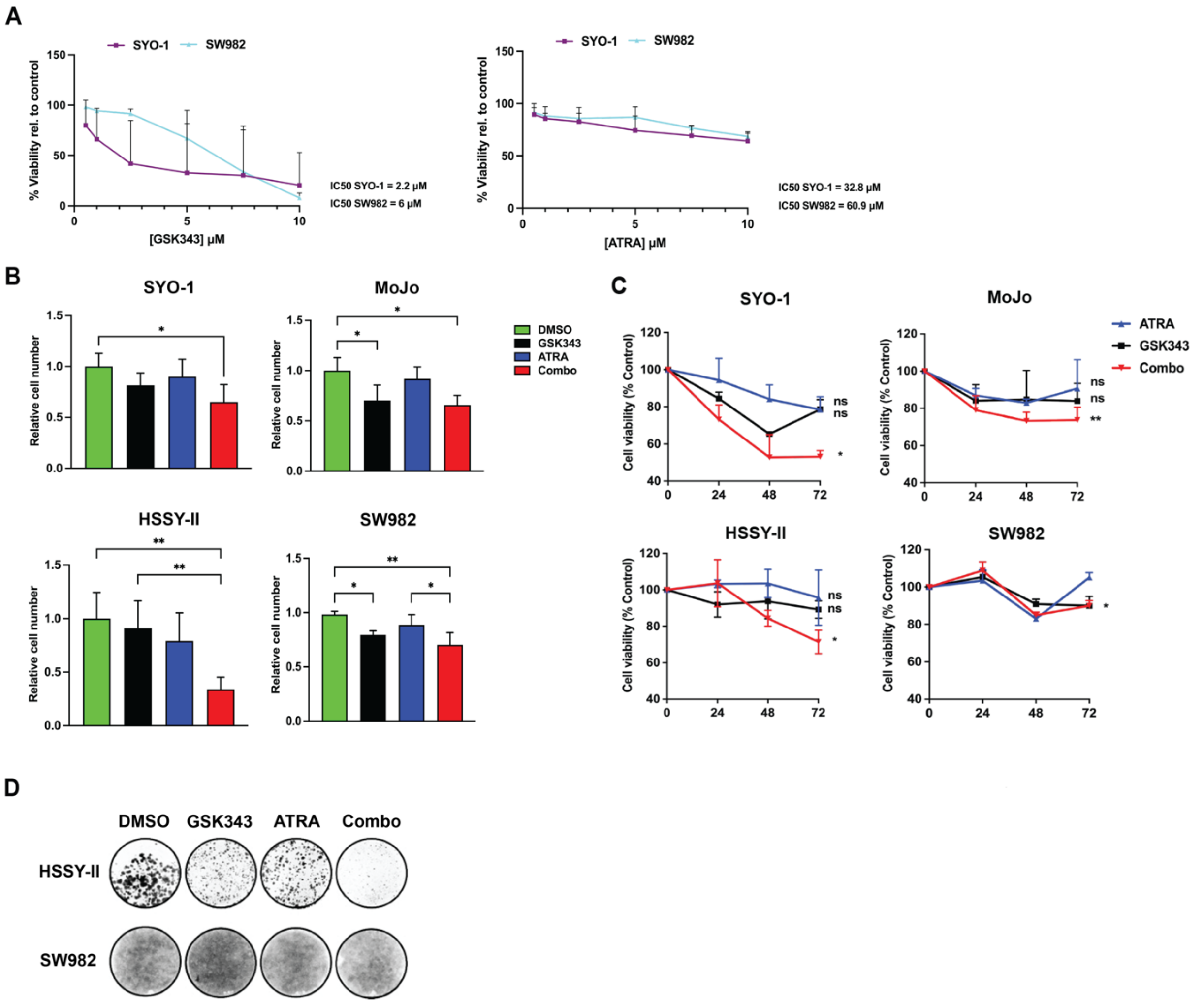

Fig. S5. Viability and colony formation assays in SS cells after treatment with GSK343 and ATRA. **(A)** IC_{50} of GSK343 (left panel) and ATRA (right panel) after seven days treatment in SYO-1 (TP-SS) and SW982 (TN-SS), as assessed by BrdU proliferation assay. **(B)** Relative cell number of SYO-1, MoJo, HSSY-II and SW982 cells after treatment with DMSO, GSK343, ATRA, or the GSK343+ATRA combination for three days. Statistical analysis: one-way ANOVA with multiple comparison tests from at least three independent experiments. **(C)** MTT-based cell viability assay. MoJo, SYO-1, HSSY-II, and SW982 cells were treated with DMSO, 2.5 µM GSK343, or 5 µM ATRA, alone or in combination. MTT-based cell viability assay was performed after 24, 48, and 72 h. The percent of viable to DMSO treated cells are presented. Experiments were repeated at least three times. Statistical analysis: multiple regression analysis. **(D)** Translocation positive HSSY-II SS cells and translocation negative SW982 SS cells were treated with DMSO, GSK343 or ATRA individually as well as in combination (Combo). Colonies were stained with crystal violet after four weeks. Representative plates from three individual experiments are shown. Figure corresponds to Figure 4.

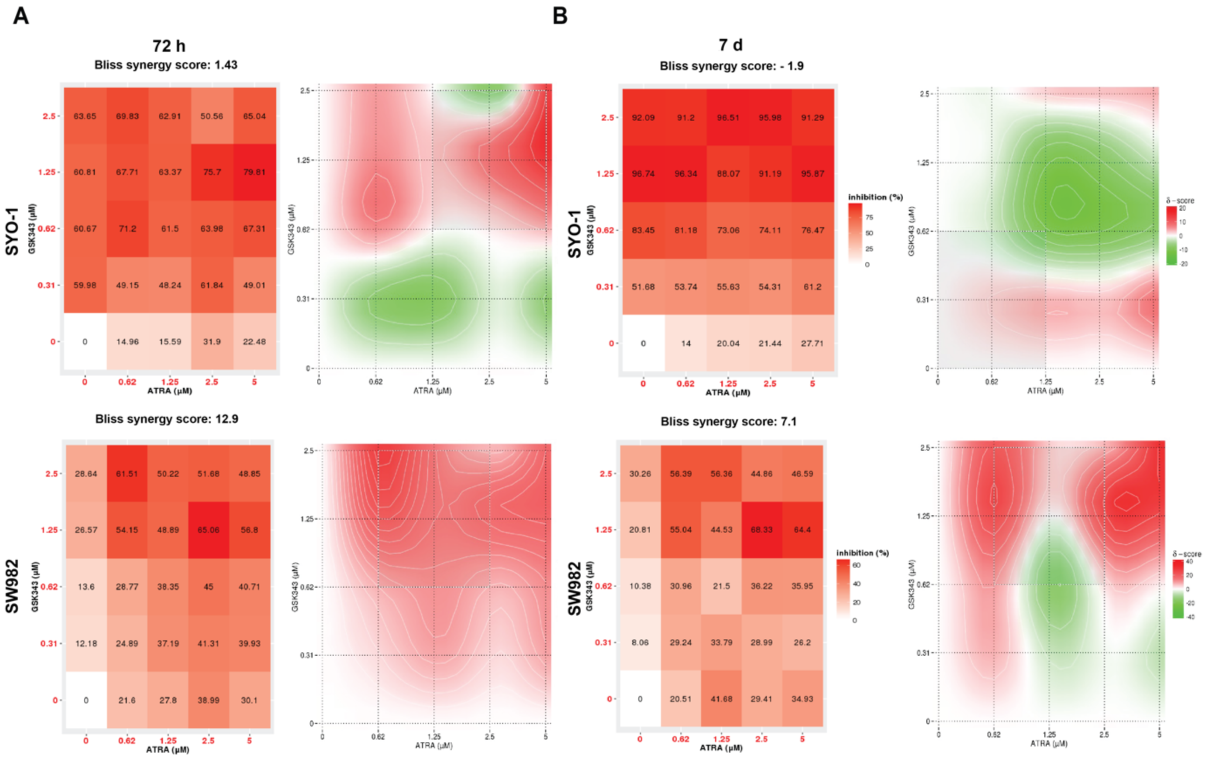

Fig. S6. Combinatorial treatments of GSK343 and ATRA in SS cells. (A, B) Inhibition matrix in %, and synergy plots from 72 h (A) or seven days (B) treatment in SYO-1 and SW982 cells with different concentrations of GSK343 and ATRA as indicated. Bliss synergy score is specified for each cell line and timepoint.

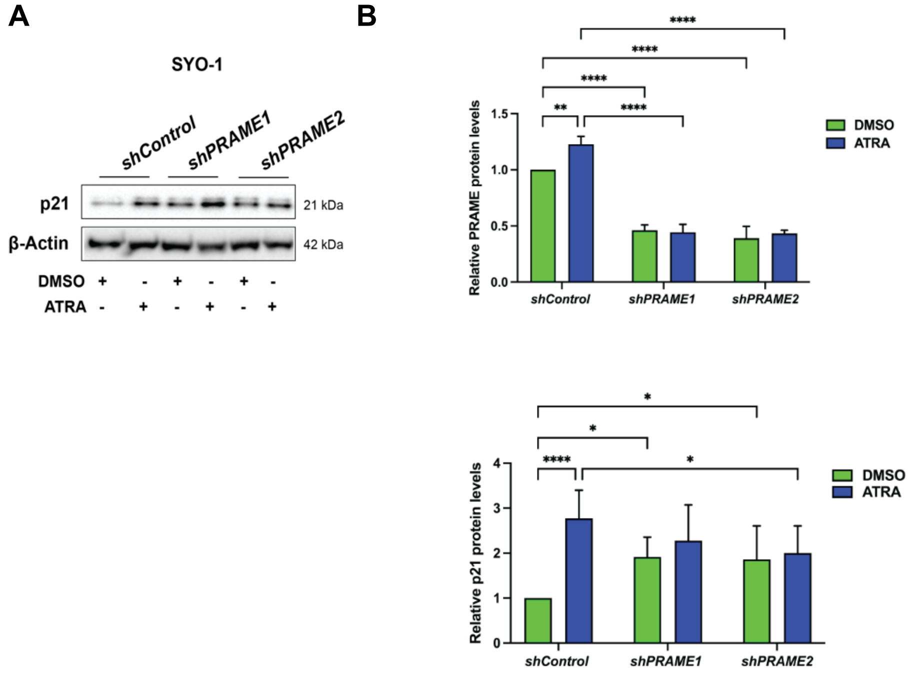

Fig. S7. PRAME and p21 protein levels in *shControl* and *shPRAME* cell lines. Cells were transduced with lentiviral particles loaded with control vector or vectors carrying either of the two *PRAME* shRNAs. **(A)** Protein levels of p21 in stable cell lines carrying the control vector or either of the two *PRAME* shRNAs. β-Actin was used as loading control. Molecular weight markers in kDa are shown to the right of each blot. Uncropped scan shown in Original data. **(B)** Quantification of PRAME (upper graph) and p21 (lower graph) levels from three independent experiments. Statistical analysis: two-way ANOVA. Data correspond to Fig. 5 (PRAME) and S7A (p21).

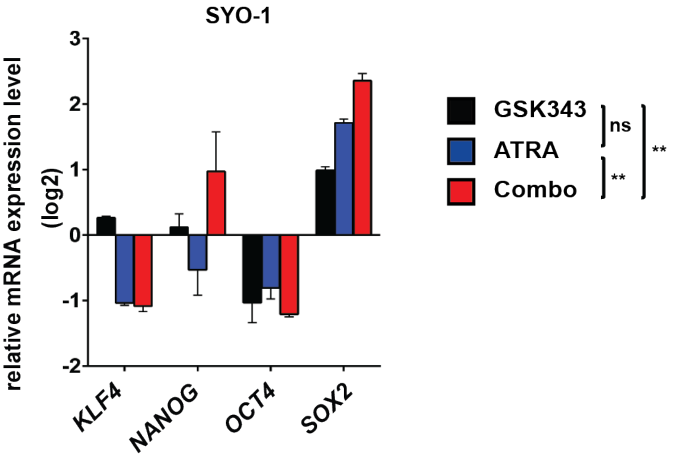

Fig. S8. Effects on RA target genes after GSK343 and ATRA treatment. SYO-1 cells were treated with 2.5 µM GSK343 and 5 µM ATRA for one week and mRNA expression of a panel of genes was assessed by RT-qPCR using *GAPDH*, β-*Actin,* or *TBP* as endogenous controls. Three independent experiments were performed, statistical analysis: two-way ANOVA. This Figure corresponds to main Fig. 6A. Raw values are presented in Original data.

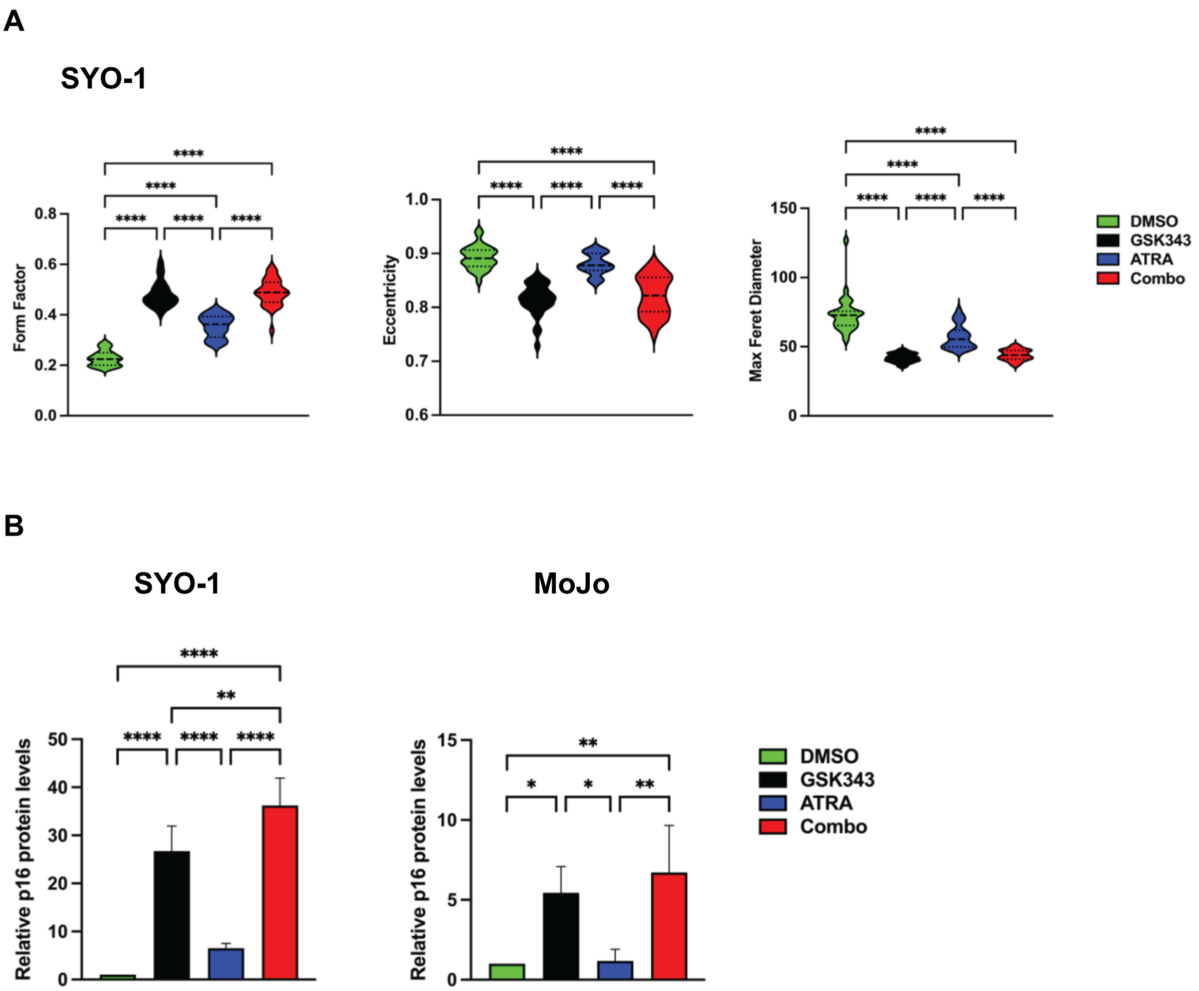

Fig. S9. Senescence parameters after treatment of SS cells. (A) SYO-1 β-gal image analysis from Figure 7B using CellProfiler. Form factor (Circular object = 1), eccentricity (circle = 0; line segment = 1), and Max Feret Diameter [Distance between the center of an object (cell) and the most distant point] are shown. Statistical analysis: one-way ANOVA. **(B)** Quantification of the data shown in main Fig. 7D. The graphs represent the quantification of Western blots obtained from at least three independent experiments and one way ANOVA test was performed for statistical significance.

Table S1. Detailed Enrichment Results of the gene sets. Enrichment results for the gene sets used. Size: Number of genes in the gene set; ES: Enrichment score for the gene set; NES: Normalized enrichment score; NOM *p*-value: Nominal *p*-value, *i.e.* the statistical significance of the enrichment score; FDR *q*-value: False discovery rate; FWER *p*-value: Familywise-error rate; RANK AT MAX: rank at which the maximum enrichment score was observed in the list; LEADING EDGE: the three statistical parameters which define the leading edge subset *i.e.* tag, list, and signal. Data corresponds to the GSEA graphs shown in main Figure 2 and Figure S3.

NAME	SIZE	ES	NES	NOM p-val	FDR q-val	FWER p-val	RANK AT MAX	LEADING EDGE
DELACROIX_RAR_BOUND_ES	411	-0,36070648	-1,44608	0,005870841	0,005870841	0,003	3745	tags=24%, list=20%, signal=29%
GOBP_RIBOSOME_BIOGENESIS	278	0,48705575	2,026907	0,001930502	0,001930502	0,001	7258	tags=64%, list=38%, signal=103%
GOBP_DNA_REPAIR	508	0,4581109	2,003233	0,001988072	0,001988072	0,001	6111	tags=50%, list=32%, signal=72%
GOBP_CHROMATIN_REMODELING	283	0,35792542	1,699459	0,014256619	0,014256619	0,007	5522	tags=43%, list=29%, signal=59%
REACTOME_RNA_POLYMERASE_I_TRANSCRIPTION_INITIATION	43	0,50174737	2,009852	0,003898636	0,003898636	0,002	6685	tags=56%, list=35%, signal=86%
REACTOME_CELL_CYCLE_CHECKPOINTS	241	0,5747244	1,772396	0,020876827	0,020876827	0,01	3604	tags=37%, list=19%, signal=45%
REACTOME_REGULATION_OF_TP53_ACTIVITY_THROUGH_PHOSPHC	90	0,5325751	1,811702	0,00998004	0,00998004	0,005	5090	tags=50%, list=27%, signal=68%

Table S2. Cell lines employed. Information on cell name, origin, and source.

Cell line	Origin	Source
SYO-1	Biphasic Synovial Sarcoma	Prof. Akira Kawai<br>National Cancer Center Hospital, Tokyo, Japan.
MoJo	Monophasic Synovial sarcoma	Dr. K. Jones,<br>Huntsman Cancer Institute, Salt Lake City, USA.
HSSY-II	Monophasic Synovial Sarcoma	Dr. Hiroshi Sonobe<br>Kochi Medical School, Nankoku, Japan.
SW982	Biphasic Synovial Sarcoma	Prof. K. Lehti, Norwegian University of Science and Technology (NTNU), Trondheim, Norway / Karolinska Institutet, Stockholm, Sweden.
RH-30	Rhabdomyosarcoma	Prof. Janet Shipley<br>The Institute of Cancer Research, London, UK.
SW872	Liposarcoma	Prof. K. Lehti, Norwegian University of Science and Technology (NTNU), Trondheim, Norway / Karolinska Institutet, Stockholm, Sweden.
RD-ES	Ewing sarcoma	Prof. Janet Shipley<br>The Institute of Cancer Research, London, UK.
SK-UT-1	Leiomyosarcoma	Prof. K. Lehti, Norwegian University of Science and Technology (NTNU), Trondheim, Norway / Karolinska Institutet, Stockholm, Sweden.

Table S3. List of Antibodies used in Western blot assays. Information on antibody, dilution, catalog number, and manufacturer.

Antibody	Dilution	Catalog number	Manufacturer
Rabbit anti SS18-SSX	1:1000	70929	Cell Signaling Technology, Leiden, Netherlands.
Mouse anti PRAME	1:500	sc-137188	Santa Cruz Biotechnology, Dallas, TX, USA.
Mouse anti Tubulin β-III (2G10)	1:2000	ab78078	Abcam, Cambridge, UK.
Rabbit anti SOX9	1:1500	sc-20095	Santa Cruz Biotechnology, Dallas, TX, USA.
Mouse anti c-MYC (9E10)	1:1000	sc-40	Santa Cruz Biotechnology, Dallas, TX, USA.
Rabbit anti p21	1:3000	12D1	Cell Signaling Technology, Leiden, Netherlands.
Rabbit anti p21	1:1000	sc-397	Santa Cruz Biotechnology, Dallas, TX, USA.
Rabbit anti p16	1:1000	E6N8P	Cell Signaling Technology, Leiden, Netherlands.
Mouse anti β-Actin	1:3000	sc-47778	Santa Cruz Biotechnology, Dallas, TX, USA.
Mouse anti GAPDH	1:3000	sc-32233	Santa Cruz Biotechnology, Dallas, TX, USA.
Rabbit anti EZH2 (D2C9)	1:2000	5246S	Cell Signaling Technology, Leiden, Netherlands.
Mouse anti PPARγ	1:1000	sc-7273	Santa Cruz Biotechnology, Dallas, TX, USA.
Mouse anti RARα	1:500	sc-515796	Santa Cruz Biotechnology, Dallas, TX, USA.
Mouse anti Histone H3K27me3	1:2000	61017	Active Motif, Carlsbad, CA, USA.
Rabbit anti Histone H3	1:2000	ab1791	Abcam, Cambridge, UK.

Table S4. List of Antibodies used in immunofluorescence (IF) and Proximity Ligation Assays (PLAs). Information about antibody, dilution, catalog number, and manufacturer. Only one dilution specified when the same antibody dilution was used in both assays.

**Antibody**	**Dilution IF/PLA**	**Catalog number**	**Manufacturer**
Mouse anti PRAME	1:200	sc-137188	Santa Cruz Biotechnology, Dallas, TX, USA
Mouse anti Tubulin β-III (2G10)	1:2000	ab78078	Abcam, Cambridge, UK
Rabbit anti Retinoic acid Receptor alpha	1:200/1:250	PA1-810A	Thermo Fisher Scientific, Waltham, MA, USA
Rabbit anti EZH2 (D2C9)	1:200	5246S	Cell Signaling Technology, Leiden, Netherlands

Supplementary References

1. Du N, Bao W, Zhang K, Lu X, Liu G, Zhang C, et al. Molecular Cytogenetic Characterization of Synovial Sarcoma Cell Line SW982. Nov. Approaches Cancer Study 2021;6:606–13.

2. Chang JH, Lee KJ, Kim SK, Yoo DH, Kang TY. Validity of SW982 synovial cell line for studying the drugs against rheumatoid arthritis in fluvastatin-induced apoptosis signaling model. Indian J. Med. Res. 2014;139:117–24.

3. Brashears CB, Prudner BC, Rathore R, Caldwell KE, Dehner CA, Buchanan JL, et al. Malic Enzyme 1 Absence in Synovial Sarcoma Shifts Antioxidant System Dependence and Increases Sensitivity to Ferroptosis Induction with ACXT-3102. Clin. Cancer Res. 2022;28:3573–89.

4. Henderson SR, Guiliano D, Presneau N, McLean S, Frow R, Vujovic S, et al. A molecular map of mesenchymal tumors. Genome Biol. 2005;6.

5. Filion C, Motoi T, Olshen AB, Laé M, Emnett RJ, Gutmann DH, et al. The EWSRI/NR4A3 fusion protein of extraskeletal myxoid chondrosarcoma activates the PPARG nuclear receptor gene. J. Pathol. 2009;217:83–93.

6. Tate JG, Bamford S, Jubb HC, Sondka Z, Beare DM, Bindal N, et al. COSMIC: The Catalogue Of Somatic Mutations In Cancer. Nucleic Acids Res. 2019;47:D941–7.

7. Network TCGAR. Comprehensive and Integrated Genomic Characterization of Adult Soft Tissue Sarcomas. Cell 2017;171:950–65.

8. de Bruijn I, Kundra R, Mastrogiacomo B, Tran TN, Sikina L, Mazor T, et al. Analysis and Visualization of Longitudinal Genomic and Clinical Data from the AACR Project GENIE Biopharma Collaborative in cBioPortal. Cancer Res. 2023;83:3861–7.

9. Cerami E, Gao J, Dogrusoz U, Gross BE, Sumer SO, Aksoy BA, et al. The cBio Cancer Genomics Portal: An open platform for exploring multidimensional cancer genomics data. Cancer Discov. 2012;2:401–4.

10. Chen Y, Su Y, Cao X, Siavelis I, Leo IR, Zeng J, et al. Molecular Profiling Defines Three Subtypes of Synovial Sarcoma. Adv. Sci. 2024;2404510:1–18.

11. Roszik J, Wang WL, Livingston JA, Roland CL, Ravi V, Yee C, et al. Overexpressed PRAME is a potential immunotherapy target in sarcoma subtypes. Clin. Sarcoma Res. 2017;7:1–7.

12. Ritchie ME, Phipson B, Wu D, Hu Y, Law CW, Shi W, et al. Limma powers differential expression analyses for RNA-sequencing and microarray studies. Nucleic Acids Res. 2015;43:e47.

13. Yu G, Wang LG, Han Y, He QY. ClusterProfiler: An R package for comparing biological themes among gene clusters. Omi. A J. Integr. Biol. 2012;16:284–7.

14. Liberzon A, Birger C, Thorvaldsdóttir H, Ghandi M, Mesirov JP, Tamayo P. The Molecular Signatures Database Hallmark Gene Set Collection. Cell Syst. 2015;1:417–25.

15. Ianevski A, Giri AK, Aittokallio T. SynergyFinder 3.0: an interactive analysis and consensus interpretation of multi-drug synergies across multiple samples. Nucleic Acids Res. 2022;50:739–43.

16. Stirling DR, Swain-Bowden MJ, Lucas AM, Carpenter AE, Cimini BA, Goodman A. CellProfiler 4: improvements in speed, utility and usability. BMC Bioinformatics 2021;22:1–11.

17. Mushtaq M, Kovalevska L, Darekar S, Abramsson A, Zetterberg H, Kashuba V, et al. Cell stemness is maintained upon concurrent expression of RB and the mitochondrial ribosomal protein S18-2. Proc. Natl. Acad. Sci. U. S. A. 2020;117:15673–83.
